# Genetics and breeding of phenolic content in tomato, eggplant and pepper fruits

**DOI:** 10.3389/fpls.2023.1135237

**Published:** 2023-03-21

**Authors:** Elena Rosa-Martínez, Arnaud Bovy, Mariola Plazas, Yury Tikunov, Jaime Prohens, Leandro Pereira-Dias

**Affiliations:** ^1^ Instituto de Conservación y Mejora de la Agrodiversidad Valenciana, Universitat Politècnica de València, Valencia, Spain; ^2^ Plant Breeding, Wageningen University & Research, Wageningen, Netherlands; ^3^ Faculdade de Ciências, Universidade do Porto, Porto, Portugal

**Keywords:** phenylpropanoid pathway, QTLs, structural and regulatory genes, flavonoids, phenolic acids, breeding strategies, transcription factors, polyphenols

## Abstract

Phenolic acids and flavonoids are large groups of secondary metabolites ubiquitous in the plant kingdom. They are currently in the spotlight due to the numerous health benefits associated with their consumption, as well as for their vital roles in plant biological processes and in plant-environment interaction. Tomato, eggplant and pepper are in the top ten most consumed vegetables in the world, and their fruit accumulation profiles have been extensively characterized, showing substantial differences. A broad array of genetic and genomic tools has helped to identify QTLs and candidate genes associated with the fruit biosynthesis of phenolic acids and flavonoids. The aim of this review was to synthesize the available information making it easily available for researchers and breeders. The phenylpropanoid pathway is tightly regulated by structural genes, which are conserved across species, along with a complex network of regulatory elements like transcription factors, especially of MYB family, and cellular transporters. Moreover, phenolic compounds accumulate in tissue-specific and developmental-dependent ways, as different paths of the metabolic pathway are activated/deactivated along with fruit development. We retrieved 104 annotated putative orthologues encoding for key enzymes of the phenylpropanoid pathway in tomato (37), eggplant (29) and pepper (38) and compiled 267 QTLs (217 for tomato, 16 for eggplant and 34 for pepper) linked to fruit phenolic acids, flavonoids and total phenolics content. Combining molecular tools and genetic variability, through both conventional and genetic engineering strategies, is a feasible approach to improve phenolics content in tomato, eggplant and pepper. Finally, although the phenylpropanoid biosynthetic pathway has been well-studied in the Solanaceae, more research is needed on the identification of the candidate genes behind many QTLs, as well as their interactions with other QTLs and genes.

## 1 Introduction

The Solanaceae family includes some of the world most important crops (Olmstead et al., 2008). Among them, tomato (*Solanum lycopersicum* L.), eggplant (*S. melongena* L.) and pepper (*Capsicum annuum* L.) are widely cultivated worldwide for their edible berries, providing a wealth of nutritional and health-promoting compounds to the human diet.

Over the last decades, we have witnessed remarkable developments in plant breeding, particularly regarding yield, pest and disease resistance, and fruit external quality. However, traits like flavor and nutritional quality have been neglected for the sake of higher yields and longer shelf-life (Tieman et al., 2017; Zhu et al., 2018). With the increasing amount of data linking a lower risk of disease incidence to a regular consumption of vegetables, consumers are shifting towards healthier foods. In this context, plant secondary metabolites have gained a lot of attention due to their bioactive role in the human body (Kaushik et al., 2016; Pott et al., 2019).

Phenolic compounds are a large and diverse group of secondary metabolites ubiquitous in the plant kingdom. They share an aromatic ring backbone with one or several hydroxyl groups or other substitutes, such as sugar molecules or organic acids (Vogt, 2010). Depending on the number of aromatic rings and on the structural elements that bind these rings together, phenolic compounds may fall into one of several classes: phenolic acids, flavonoids, tannins, stilbenes, lignans, coumarins, chromones and xanthones (Vogt, 2010; Pott et al., 2019). Herein we focused on two major groups, phenolic acids and flavonoids, as they are the most relevant in fruits of tomato, eggplant and pepper. Within the flavonoids we have intentionally left out anthocyanins, a major group of polyphenolic pigments with numerous implications in both plant metabolism and human health, hence an important target for breeders, due to the fact that they have been comprehensively discussed in a recent review regarding their biological function and genetic regulation in the Solanaceae fruits (Liu et al., 2018).

**Figure 1:**
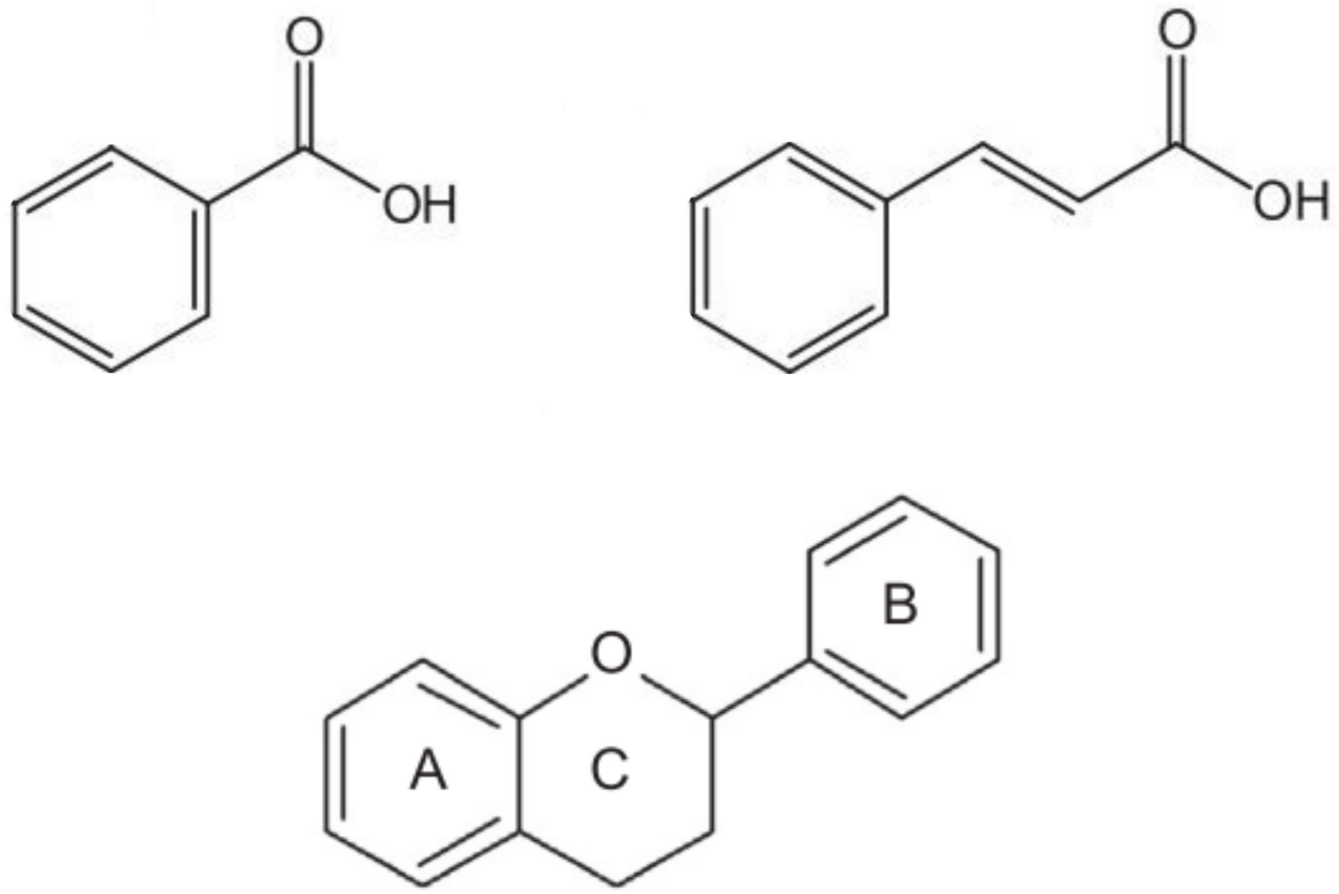
Basic structure of benzoic acids (top left), which has a carboxylic group attached to the aromatic ring (C6-C1 backbone), cinnamic acids (top right), which has a propanoic acid attached to the aromatic ring (C6-C3 backbone), and flavonoids (bottom), consisting of a benzo-γ-pyrone ring **(A)** and a phenyl ring **(B)** bound by an oxygen-containing γ-pyrone ring **(C)**.

Phenolic acids share a benzene ring and a carboxyl group as a backbone and can be classified into derivatives of benzoic acid or cinnamic acid, depending on whether the aromatic ring has a carboxylic group (C6-C1 structure) or a propanoic acid (C6-C3 structure) attached to it, respectively (**Figure 1**). Different levels of hydroxylation and methoxylation of each basic structure will result in different compounds with different antioxidant capacity (Scarano et al., 2020). Hence, within the hydroxybenzoic acid derivatives, the most representative acids are gallic, ellagic, protochatechuic, *p*-hydroxybenzoic, vanillic and syringic. On the other hand, the major hydroxycinnamic acids are caffeic, ferulic, sinapic and *p*-coumaric acids, and their derivatives (Valanciene et al., 2020). Rather than in their free form, these compounds are usually found esterified with other organic acids (e.g., quinic, tartaric) or carbohydrates. The most relevant group of these conjugates are the esters of hydroxycinnamic acids with quinic acid, commonly called chlorogenic acids (Clifford et al., 2017). Chlorogenic acids, in particular 5-O-caffeoylquinic acid, along with other caffeic acid derivatives, constitute the most abundant type of phenolic acids in tomato, eggplant and pepper (Whitaker and Stommel, 2003; Marín et al., 2004; Slimestad and Verheul, 2009; Luthria et al., 2010; García-Valverde et al., 2013). In general, hydroxycinnamic acids are more abundant than hydroxybenzoic derivatives in plants, including the Solanaceae (Valanciene et al., 2020).

Flavonoids are the most diverse class of phenolics (Ku et al., 2020). All flavonoids share a C6-C3-C6 carbon backbone formed by a benzo-γ-pyrone structure (ring A) and a phenyl ring (ring B) bound by an oxygen-containing γ-pyrone ring (ring C), as shown in **Figure 1**. Based on the position at which the B ring is attached to the C ring, as well as the oxidation and saturation degree of the heterocyclic C ring, flavonoid molecules are classified into flavones, flavonols, flavanones, anthocyanidins, flavanols or isoflavones (Vogt, 2010; Ku et al., 2020). Structural variations and the ability to be modified enzymatically are the main reasons contributing to their unique properties and broad functional diversity (Vogt, 2010; Ku et al., 2020). Furthermore, flavonoids antioxidant capacity varies depending on the number and position of hydroxyl groups in the catechol B ring and their position on the pyran C ring, and with the presence of other functional groups on the molecule and their arrangement around the nuclear structure (Dias et al., 2021).

Phenolic acids and flavonoids have been extensively characterized in tomato, eggplant and pepper fruits of cultivated varieties and wild relatives. Although these vegetables belong to the same family, their accumulation profile is substantially different (Rosa-Martínez et al., 2021).

### 1.1 Why breeding for higher levels of phenolic acids and flavonoids in tomato, eggplant and pepper?

Numerous health benefits have been linked to a phenolic-rich diet, namely related to both the antioxidant properties of phenolic compounds, which translate into their direct ability of reducing oxidant species, scavenging free radicals and chelating metal ions, and to their ability to modulate intracellular signaling molecules/pathways (Williams et al., 2004; Galano et al., 2016; Cosme et al., 2020). Thus, several studies have reported phenolic compounds to show anti-inflammatory, anti-microbial, anti-diabetic, anti-tumoral, and cardioprotective properties *in vitro* and in human trials. For instance, a major circulating human metabolite of quercetin has been proved to decrease the transcription of genes encoding pro-inflammatory interleukins and enzymes involved in oxidative stress responses, thus reducing the effects of atherosclerosis (Derlindati et al., 2012). Gallic acid had a potent effect on Herpes simplex virus type 1 and parainfluenza type 3 (Özçelik et al., 2011). The flavanones naringin and naringenin have been reported to enhance the expression of the insulin receptor GLUT4 and adiponectin in type II diabetic rats (Ahmed et al., 2017). Caffeic acid has demonstrated anti-proliferative and apoptotic effects on human melanoma, and colon, breast and liver cancer cells (Weng and Yen, 2012; Pelinson et al., 2019; Santana-Gálvez et al., 2020). Luteolin has been proven to prevent ischemia-reperfusion injury by reducing necrosis and apoptosis in rat cardiomyocytes and to induce arterial relaxation (Luo et al., 2017). Lastly, human cohorts have shown that chlorogenic acid (25-400 mg d^-1^) may significantly reduce blood pressure in mild hypertensive adults (Mubarak et al., 2012; Kajikawa et al., 2019).

Furthermore, phenolics are important to an array of plant biological processes and to plant-environment interaction, such as protection against solar radiation, pathogens, herbivores and mechanical damage, attracting pollinators and seed dispersers, and abiotic stress signaling. Flavonoids are essential for male fertility in many species, so that they are found as part of the pollen grain wall structure (Wang et al., 2020; Dong and Lin, 2021). Down-regulation of the flavonoid biosynthesis has been shown to produce defective seeds due to impaired pollen-tube growth in tomato (Schijlen et al., 2007). Flavonols participate in the pollen’s response to heat stress through scavenging of reactive oxygen species (ROS), thus maintaining its reproductive success (Muhlemann et al., 2018; Rutley et al., 2021). Flavonoids are abundantly present in the tomato fruit cuticle and epidermal cells. Low expression of a flavonoid biosynthesis key enzyme resulted in lower water permeability, lower stiffness and increased deformation of the cuticle (España et al., 2014; Heredia et al., 2015). Zhang et al. (2013) observed that anthocyanins-pigmented tomatoes had extended shelf life compared to anthocyanin-free ones, likely due to their antioxidant and ROS signaling properties. Levels of quercetin-O-rhamnoside-O-hexoside and chlorogenic acid remarkably increased in response to chilling injury, and induced stress tolerance in pepper (López-Velázquez et al., 2020). In *Arabidopsis thaliana*, flavonols have been reported to act as positional signals, integrating hormonal and ROS pathways to regulate root growth direction and rate in response to light (Silva-Navas et al., 2016; Tohge and Fernie, 2016). Also, the accumulation of anthocyanins in eggplant peel is induced by light exposure (Li et al., 2018a). In this respect, the photoprotective role against UV-B radiation of some phenolic compounds, such as anthocyanins, flavones, caffeic acid and chlorogenic acid have been widely demonstrated in several species (Sytar et al., 2018; Righini et al., 2019). Likewise, flavonoids and phenolic acids may act as a physical or chemical barrier to prevent invasion, or as a direct weapon against microbes and insects (Ramaroson et al., 2022). In tomato leaves, increased accumulation of phenolic acids was detected in response to *Pseudomonas syringae* infection (Dadáková et al., 2020), while higher accumulation of naringenin was associated with the resistance against *Botrytis cinerea* (Szymański et al., 2020).

All in all, considering that tomato, eggplant, and pepper are amongst the ten most consumed vegetables in the world, enhancing their phenolics content would lead to a promotion of the consumers health, while at the same time generating more resilient crops. In addition, the fact that some flavonoids lead to fruit pigmentation enables the development of new attractive colors, such as purple, generally associated with healthier produce, leading to diversification of the vegetable market. Finally, an increase in flavonoids content would improve postharvest quality and shelf-life. Consequently, bringing benefits to all the players in the production chain from farmers to consumers.

### 1.2 Which phenolic acids and flavonoids are found in the fruits of these crops?

In general, tomato accumulates more flavonoids than phenolic acids. The latter are mainly represented by caffeic acid and chlorogenic acid (Luthria et al., 2006; Hallmann and Rembialkowska, 2012; Martí et al., 2018), although gallic, ferulic, *p*-coumaric acids, and other derivatives, have been also identified in variable amounts in both the peel and pericarp (Rigano et al., 2014; Alarcón-Flores et al., 2016; Martí et al., 2018). Regarding flavonoids, tomato is known to be a good dietary source of naringenin chalcone and the flavonols rutin (quercetin-rutinoside), kaempferol-rutinoside and quercetin-trisaccharide (Muir et al., 2001; Slimestad et al., 2008), along with many other conjugated forms (Moco et al., 2006; Iijima et al., 2008; Alarcón-Flores et al., 2016). Within the tomato cultivated germplasm, flavonoids are accumulated almost exclusively in the fruit peel, while only trace amounts can be found in the fruit pericarp (Muir et al., 2001; Willits et al., 2005). Naringenin chalcone is the most abundant flavonoid in tomato, while rutin and kaempferol are accumulated at much lower amounts (Muir et al., 2001; Slimestad et al., 2008). Naringenin chalcone is also the pigment responsible for the transient yellow coloration of tomato fruit peel at breaker stage, during the transition from mature green to red ripe fruits (Adato et al., 2009; Ballester et al., 2010). It is worth mentioning that although cultivated tomato fruits usually do not accumulate anthocyanins, nowadays, we can find purple tomato fruits due to the introgression of wild genes (Povero et al., 2011; Colanero et al., 2020).

Eggplant is one of the vegetables with the highest antioxidant capacity. Morales-Soto et al. (2014) ranked it among the top 5 fruits and vegetables out of 44 evaluated, in terms of total antioxidant capacity. Eggplant antioxidant capacity is empirically attributed to the phenolic acid profile in the fruit flesh, and especially to its high chlorogenic acid content, but also to the presence of anthocyanins in the purple fruit peel (Docimo et al., 2016; Rosa-Martínez et al., 2021). Some have also reported the presence of flavonol derivatives in the eggplant fruit peel. In this way, Singh et al. (2017) identified 11 flavonols, including different glycosides of quercetin, kaempferol and myricetin. Docimo et al. (2016) and Sulli et al. (2021) also quantified rutin in the fruit peel of a RIL population. However, only trace amounts were detected. The first comprehensive characterization of the eggplant phenolic acid profile was reported by Whitaker and Stommel (2003). The authors identified and quantified 14 major hydroxycinnamic acid conjugates in the fruits of seven commercial cultivars. All the compounds identified were esters of caffeic acid with quinic acid and derivatives. Since then, others have evaluated the profile of phenolic acids in fruits of cultivated and wild eggplant (Singh et al., 2009; Mennella et al., 2010; Sunseri et al., 2010; Mennella et al., 2012; Prohens et al., 2012; Mori et al., 2013; Plazas et al., 2014; Stommel et al., 2015). All demonstrated that chlorogenic acid is the most abundant phenolic in eggplant fruits. Indeed, within the cultivated pool, it typically represents 80-95% of the total phenolic acids content present in the fruit flesh.

Pepper has also been reported to have high antioxidant capacity, mainly due to its outstanding content in vitamin C (Morales-Soto et al., 2014). Nevertheless, phenolics of all classes have been identified and quantified in different varieties of pepper. Recently, Lemos et al. (2019) reviewed the data regarding pepper phenolic content and compiled it in a publication, making it readily accessible. Among hydroxybenzoic acids, the major compounds reported in pepper were gallic and vanillic acids (Mokhtar et al., 2015; Mudrić et al., 2017; Moreno-Ramírez et al., 2018) and the most abundant hydroxycinnamic acid was generally chlorogenic acid (Hallmann and Rembialkowska, 2012; Fratianni et al., 2020), although *p-*coumaric, caffeic, ferulic, and sinapic acids, as well as several of their glycosides were also quantifiable (Marín et al., 2004; Mudrić et al., 2017; Fratianni et al., 2020). Regarding flavonoids, the most represented subfamilies in pepper fruits are flavonols and flavones (Liu et al., 2020). Within the first group, quercetin and its glycosides are the most common, followed by myricetin and kaempferol, while within flavones, luteolin and its derivatives are the most representative, followed by apigenin and its derivatives (Jeong et al., 2011; Wahyuni et al., 2011; Hallmann and Rembialkowska, 2012; Chen and Kang, 2013; Morales-Soto et al., 2013; Mokhtar et al., 2015; Fratianni et al., 2020; Ribes-Moya et al., 2020). In addition, cultivated pepper fruits may accumulate anthocyanins during the unripe stages, although they often degrade during the ripening process (Aza-González et al., 2013).

### 1.3 Genetic and genomic resources impact in phenolic acids and flavonoids studies

Recent advances in omics have enhanced the knowledge on the genetics of complex traits like fruit quality. Notwithstanding, not all crops have the same resources available. Tomato has been a model organism for basic and applied plant research for many years now, especially as a model for other Solanaceae. Consequently, numerous genetic and genomic tools have been developed for tomato since the late 1980’s, such as isogenic mutant libraries (Menda et al., 2004) and several intra and interspecific mapping populations (Eshed and Zamir, 1995; Monforte and Tanksley, 2000). The tomato reference genome sequence was first published in 2012 for the *S. lycopersicum* ‘Heinz 1706’ cultivar (The Tomato Genome Consortium, 2012) and has since then been re-sequenced, corrected and re-annotated to the current SL4.0 version with the ITAG4.0, available at Sol Genomics Network database (https://solgenomics.net/). Furthermore, more than 900 high-quality genome sequences of cultivated tomato and its wild relatives, including a recently published pan-genome using 725 representative tomato accessions (Gao et al., 2019), have since been released.

In eggplant, the development of genomic tools started considerably later. However, in the last years, the available genomic information of eggplant has increased dramatically. Several factors have made this possible: 1) the advent of the next-generation sequencing technologies (Gebhardt, 2016); 2) the well-established synteny between the eggplant and tomato genomes (Doganlar et al., 2002; Wu et al., 2009; Gramazio et al., 2014; Rinaldi et al., 2016; Barchi et al., 2019); and, 3) the availability of intra and interspecific mapping populations with associated genetic linkage maps (Doganlar et al., 2002; Gramazio et al., 2014). The first point led to increasingly affordable whole-genome sequencing and in those terms, Hirakawa et al. (2014) published the first draft genome using ‘Nakate-Shinkuro’, a common Asian eggplant cultivar. Moreover, in the last years several high-quality eggplant genome assemblies with substantial increases of annotated genes have been released (Wei et al., 2020; Barchi et al., 2021; Li et al., 2021). This includes the first eggplant pan-genome, assembled with sequences from 26 accessions belonging to *S. melongena*, *S. incanum* and *S. insanum* (Barchi et al., 2021).

Despite its economic relevance, pepper remains a surprisingly understudied crop. For many years, pepper research relied almost entirely on F_2_ mapping populations resulting from crosses between contrasting germplasm (Lee, 2019). The first interspecific genetic linkage map was published in 1988 using RFLPs (Tanksley et al., 1988). Since then, several intra and interspecific maps have been constructed to dissect traits of interest (Lee, 2019). Thus, these first maps were of paramount importance to shed light into the synteny between pepper and tomato genomes (Rinaldi et al., 2016) and enable marker-assisted selection and QTL dissection (Wahyuni et al., 2014; Lee, 2019). Recent technological advances have made it possible for researchers to have publicly-available high-quality genome sequences. The first pepper genomes were published in 2014 with the complete sequences of *C. annuum* cv. Serrano Criollo de Morelos 334 (CM334), *C. chinense* PI159236, *C. annuum* cv. Zunla-1, and *C. annuum* var. *glabriusculum* (Kim et al., 2014; Qin et al., 2014). Since then, new genomes and improved versions of the CM334 accession have been released (Kim et al., 2017; Hulse-Kemp et al., 2018; Acquadro et al., 2020), including the first pepper pan-genome (Ou et al., 2018). Such powerful genomic resources have been successfully applied in many genome-wide association studies (Colonna et al., 2019; Siddique et al., 2019; Lee et al., 2020). However, pepper unusually large genome (~3.5Gb) and degree of repetitiveness (~80%), compared to other Solanaceae, have hampered the study of complex traits.

These tools have helped to identify QTLs and candidate genes associated with the biosynthesis of phenolic acids and flavonoids in tomato, eggplant and pepper. The aim of the following sections is to integrate and summarize the available literature, making it easily available for researchers and breeders.

## 2 Genetic basis of phenolic compounds biosynthesis and accumulation

First, a search throughout the most recent genome versions of tomato, eggplant and pepper, at the Sol Genomics platform, for annotated putative orthologue genes encoding key enzymes involved in the phenylpropanoid pathway, was conducted. For this purpose, a blastp was performed using the sequence of each verified protein from *S. lycopersicum*, or *Arabidopsis thaliana* when the former was not available, which were retrieved from the NCBI protein database (https://www.ncbi.nlm.nih.gov/protein/), and selected hits with an e-value threshold of 1·e^-10^, score >200 and identity >70%. Secondly, a literature review was carried out to compile all relevant QTLs, and associated candidate genes, related to the phenolic compounds’ biosynthesis and accumulation in Solanaceae fruits.

### 2.1 Biosynthesis of phenolic acids and flavonoids: Key annotated genes and structural enzymes of the phenylpropanoid pathway

All phenolic compounds ultimately stem from the shikimic acid pathway, which starts with phosphoenolpyruvate and D-erythrose-4-phosphate forming the C6 core aromatic ring with one carboxyl and three hydroxyl substituents (Pott et al., 2019).

**Figure 2:**
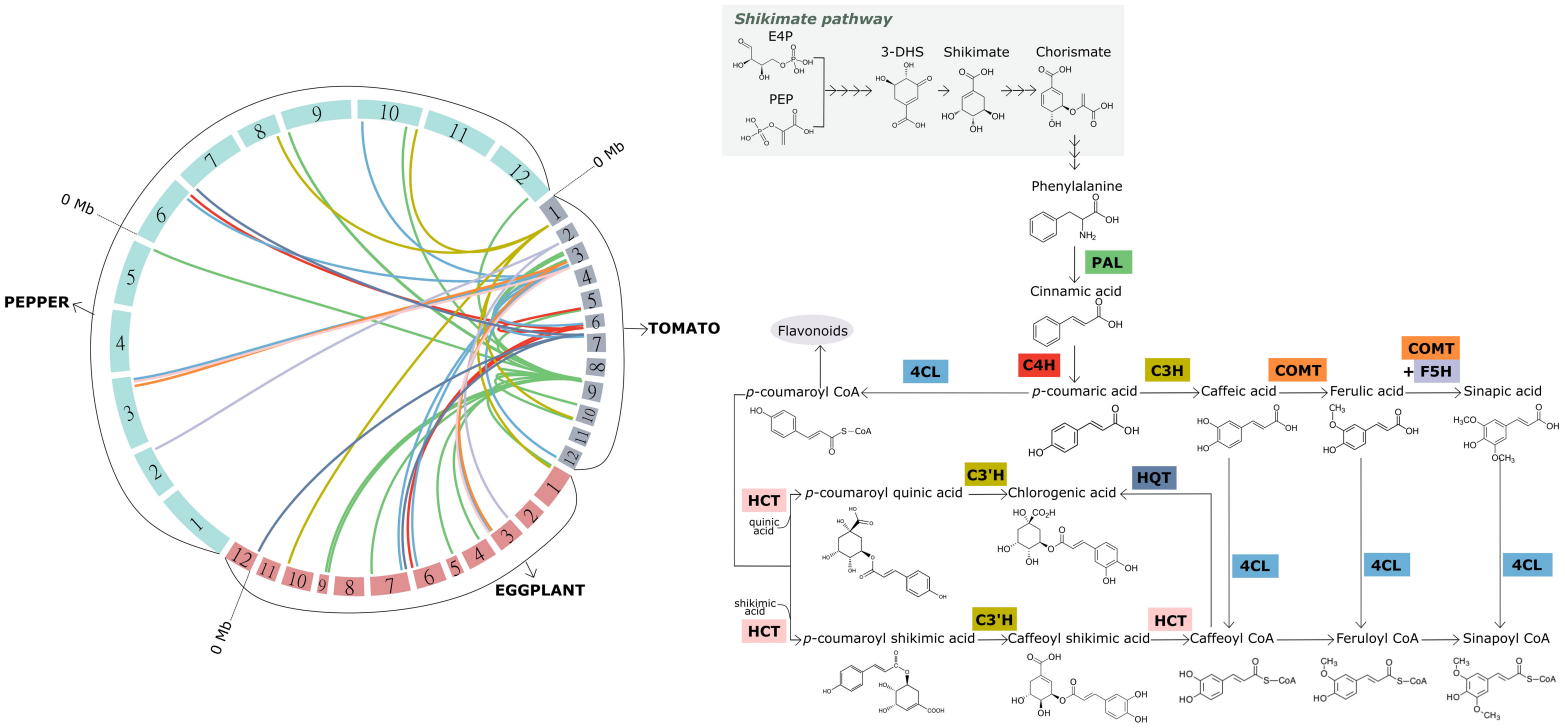
Macrosynteny among the tomato, eggplant and pepper genomes for candidate genes encoding the key enzymes involved in the synthesis of hydroxycinnamic acids (left), and overview of the aforementioned synthesis pathway (right). The 12 chromosomes of each of the three genomes represented, which are arranged to form the circle on the left, are colored differently according to the species. The chromosomes are scaled according to their size (Mb). Links with different colors inside the circle were used to connect the physical position of homologous genes within and among the genomes. Same color links represent genes encoding the same key enzyme of the pathway. The acronym of each key enzyme is indicated along the synthesis pathway (right), and framed in the same color as the corresponding link. PAL, phenylalanine ammonia-lyase; C4H, cinnamate 4-hydroxylase; 4CL, 4-coumaroyl-CoA ligase; C3H, 4-coumarate 3-hydroxylase; C3’H, p-coumaroyl ester 3′-hydroxylase; HCT, hydroxycinnamoyl-CoA:shikimate/quinate hydroxycinnamoyl transferase; HQT, hydroxycinnamoyl-CoA:quinate hydroxycinnamoyl transferase; COMT, caffeate O-methyltransferase; F5H, ferulate 5-hydroxylase (F5H). The genes used for creating the links resulted from a blastp against the current protein databases of tomato, eggplant and pepper at the Sol Genomics platform (http://www.solgenomics.net). The hits with the lowest e-value, higher sequence size and higher sequence identity (>70%) were considered.

Regarding the synthesis of hydroxybenzoic acids, two pathways have been described in plants. On one hand, the shikimate/chorismate pathway starts with two products of shikimic acid transformation: 3-dihydroshikimic acid (3-DHS) and chorismic acid. In this way, 3-DHS serves as a precursor of protocatechuic acid and gallic acid, which in turn is transformed into ellagic acid and other derivatives; while from chorismic acid *via* the intermediate isochorismic acid, salicylic acid and a wide range of dihydroxybenzoic acids (DHBA) are synthesized (Wildermuth, 2006). Alternatively, hydroxybenzoic acids can also be synthesized from phenylalanine metabolism, in this case by means of the C3 side chain shortening of the hydroxycinnamic C6-C3 structure. In this case, the benzoic acid would be synthesized from cinnamic acid, salicylic acid from *o*-coumaric acid, 4-hydroxybenzoic acid from *p*-coumaric acid, protocatechuic acid from caffeic acid, vanillic acid from ferulic acid and syringic acid from sinapic acid. A network of multiple paths has been proposed within that second pathway, although the contribution of each one remains unclear. For further information about the synthesis pathways of benzoic derived acids we’d like to refer to Widhalm and Dudareva (2015). Not all the enzymes involved in the synthesis of hydroxybenzoic acids have been identified. Nevertheless, there are several key enzymes, extensively studied in tomato, eggplant and pepper, which could be targeted to increase the production of phenolic acids (**Figure 2**; **Supplementary Data S1**).

The synthesis of phenylalanine from chorismic acid is considered the branching point that links the shikimate pathway to the phenylpropanoid pathway. The synthesis of *p*-coumaroyl-CoA from phenylalanine in a three-step reaction is considered the starting point of the synthesis of both hydroxycinnamic acids and flavonoids (Vogt, 2010). These initial steps involve three key enzymes: phenylalanine ammonia-lyase (PAL), which catalyzes the non-oxidative deamination of phenylalanine to cinnamic acid, cinnamate 4-hydroxylase (C4H), which catalyzes the subsequent formation of *p*-coumaric acid, and 4-coumaroyl-CoA ligase (4CL), which is involved in the synthesis of the next branching element along the pathway, *p*-coumaroyl-CoA (Marchiosi et al., 2020) (**Figure 2**; **Supplementary Data S1**).

From cinnamic acid, *p*-coumaric acid or *p*-coumaroyl-CoA the other major hydroxycinnamic acids (caffeic, ferulic and sinapic acids) are synthesized through a multi-step chain reaction, either by direct transformation or by their CoA-conjugated intermediates. The pool of enzymes catalyzing this series of reactions is also well characterized (Marchiosi et al., 2020). Again, three key enzymes are responsible for the biosynthesis of the following hydroxycinnamic acids: 4-coumarate 3-hydroxylase (C3H), which catalyzes the hydroxylation of *p*-coumaric acid to caffeic acid; caffeate O-methyltransferase (COMT), which is involved in both the subsequent formation of ferulic acid and, together with ferulate 5-hydroxylase (F5H), in the synthesis of sinapic acid. 4CL also catalyzes the synthesis of each hydroxycinnamic acid-CoA conjugate (**Figure 2**; **Supplementary Data S1**).

Starting from *p*-coumaroyl-CoA, two synthesis pathways have been proposed for chlorogenic acid (CGA). Again, three key enzymes catalyze these steps (Gramazio et al., 2014). In one branch, *p*-coumaroyl-CoA is converted to *p*-coumaroyl quinic acid by the enzyme hydroxycinnamoyl-CoA:shikimate/quinate hydroxycinnamoyl transferase (HCT); then, the latter compound is hydroxylated to form CGA with the action of *p*-coumaroyl ester 3′-hydroxylase (C3’H). In a second branch, *p*-coumaroyl shikimic acid is synthesized from *p*-coumaroyl-CoA *via* HCT, followed by two-step transformation to caffeoyl-CoA *via* the intermediate caffeoyl shikimic acid and catalyzed by C3’H and HCT. Finally, caffeoyl-CoA is trans-esterified with quinic acid to form CGA *via* the enzyme hydroxycinnamoyl-CoA:quinate hydroxycinnamoyl transferase (HQT) (**Figure 2**; **Supplementary Data S1**).

Efforts have been made in tomato towards the characterization of the CGA pathway genes through transgenesis, gene expression and enzymatic assays. In this way, gene silencing of an HQT-encoding gene resulted in a 98% reduction of CGA in tomato transgenic lines (Niggeweg et al., 2004), while no further soluble phenolics were affected, demonstrating that the encoded enzyme constitutes the primary route for the synthesis of CGA and controls the flux of the pathway (Niggeweg et al., 2004). More recently, several isomers of dicaffeoylquinic acids (di-CQAs), namely 1,3-, 3,4-, 1,5-, 3,5- and 4,5-di-CQA, have been identified in tomato fruits and their biosynthetic pathway has been elucidated (Moglia et al., 2014). The authors partially purified the enzyme responsible for the chlorogenate:chlorogenate transferase (CCT) activity, which was suggested to be HQT, and this role was confirmed through expression of *SlHQT* in *E. coli.* The authors comprehensively characterized the CCT activity and structural features of the HQT enzyme, as well as its subcellular localization, so that they determined the dual catalytic activity of the enzyme, which would act in the formation of CGA in the cytosol using CoA thioester as acyl donor, and as CCT in the formation of di-CQAs in the vacuole at lower pH and in the presence of high concentrations of CGA, using CGA as acyl donor (Moglia et al., 2014).

In eggplant, an interspecific anchored linkage map, based on a first backcross generation (BC_1_) of *S. melongena* ‘AN-S-26’ × *S. incanum* ‘MM577’, was developed by Gramazio et al. (2014) and exploited for locating genes involved in the CGA synthesis pathway. Based on synteny with the Tomato EXPEN-2000 genetic linkage map, the authors mapped candidate genes encoding each of the six key enzymes involved in the CGA synthesis pathway in eggplant (*PAL, C4H, 4CL, HCT, C3′H, HQT*). They were located on linkage groups E09, E06, E03, E03, E01 and E07, respectively, and showed collinearity with the corresponding genes of the tomato genetic map (Gramazio et al., 2014). Once the sequences of key genes in the chlorogenic acid synthesis pathway were known, gene expression analyses were also performed in eggplant to characterize them and to understand their tissue-specific accumulation. Thus, Docimo et al. (2016) found the higher contents of CGA in fruits, compared to other tissues, to be correlated with elevated transcript abundance of the structural genes *PAL, C4H, 4CL* and *HQT*. They also isolated putative orthologs of the two CGA biosynthetic genes, *PAL* and *HQT*, from an Occidental *S. melongena* variety and demonstrated that both differed from homologs of Asiatic varieties. In addition, using a gene expression panel composed of 15 diverse *S. melongena* landraces and eight accessions of five related species of *Solanum* subgenus *Leptostemonum*, Meyer et al. (2019) characterized the genes encoding *HCT* and *HQT.* Their results suggested that in eggplant *SmHCT* was implicated in the synthesis of mono-caffeoylquinic acid (CQA) (CGA and its isomers 3-CQA, 4-CQA, 5-*cis*-CQA), while *SmHQT* was only catalyzing di-CQA formation. The biosynthesis of flavonoids has been extensively studied, hence, most of the key elements have been identified, especially in tomato (Tohge et al., 2017; Sacco et al., 2019; Tohge et al., 2020).

**Figure 3:**
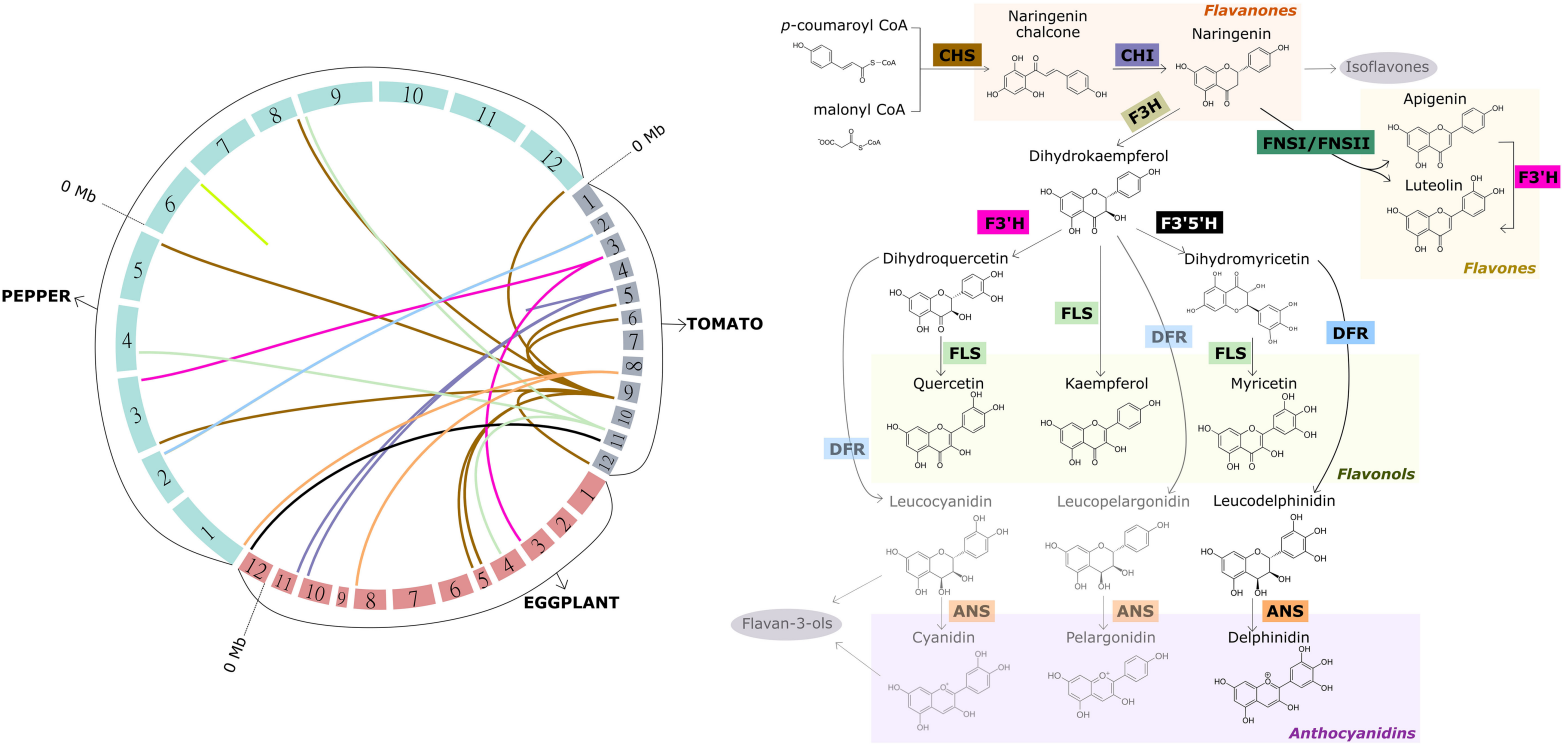
Macrosynteny among the tomato, eggplant and pepper genomes for candidate genes encoding the key enzymes involved in the synthesis of major flavonoids in fruits of these crops other than anthocyanins (left), and overview of the aforementioned synthesis pathway (right). The 12 chromosomes of each of the three genomes represented, which are arranged to form the circle on the left, are colored differently according to the species. The chromosomes are scaled according to their size (Mb). Links with different colors inside the circle were used to connect the physical position of homologous genes within and among the genomes. Same color links represent genes encoding the same key enzyme of the pathway. The acronym of each key enzyme is indicated along the synthesis pathway (right), and framed in the same color as the corresponding link. CHS, chalcone synthase; CHI, chalcone isomerase; F3H, flavanone-3-hydroxylase; F3’H, flavanone-3’-hydroxylase; F3’5’H, flavonoid-3’,5’-hydroxylase; FLS, flavonol synthase; DFR, dihydroflavonol 4-reductase; ANS, anthocyanidin synthase; FNSI/FNSII, flavone synthase I/II. The genes used for creating the links resulted from a blastp against the current protein databases of tomato, eggplant and pepper at the Sol Genomics platform (http://www.solgenomics.net). The hits with the lowest e-value, higher sequence size and higher sequence identity (>70%) were considered.

Briefly, naringenin chalcone (flavanone) is synthetized from *p*-coumaroyl-CoA and malonyl-CoA by chalcone synthase (CHS) and represents the main intermediate of flavonol biosynthesis. Naringenin chalcone is catalyzed by chalcone isomerase (CHI) and converted to the flavanone naringenin, which is a branching point for the formation of several groups of flavonoids. For example, naringenin can be converted to flavonols through the action of four key enzymes. First, flavanone-3-hydroxylase (F3H) converts naringenin into dihydrokaempferol (DHK), which is then converted into dihydroquercetin (DHQ) through the action of flavanone-3’-hydroxylase (F3’H). Finally, dihydroquercetin is catalyzed by flavonol synthase (FLS) in order to produce the flavonol quercetin. Likewise, the flavonol kaempferol is immediately synthesized from dihydrokaempferol through FLS catalysis, skipping hydroxylation at the C-3′ position (**Figure 3**; **Supplementary Data S1**).

Gene expression data of ripening tomato peel showed high levels of transcripts of most of the enzymes involved in the flavonoid biosynthesis (CHS, F3H and FLS) except for CHI, whose levels were below the detection limit (Muir et al., 2001; Bovy et al., 2002; Willits et al., 2005). This confirmed that the complete flavonoid pathway is present in the fruit peel but the regulation mechanism of CHI expression is rate-limiting the flavonol biosynthesis, probably due to a mutation in the CHI promotor sequence. In contrast, cultivated tomato flesh showed no detectable levels of any of the above-mentioned transcripts, indicating that the flavonoid biosynthesis pathway is inactive in this tissue (Muir et al., 2001; Bovy et al., 2002; Willits et al., 2005).

Furthermore, dihydrokaempferol can be hydroxylated at the 5’ position of the B-ring in addition to the 3’ position by the flavonoid-3’,5’-hydroxylase (F3’5’H), converting DHK into dihydromyricetin (DHM), which is in turn transformed into the flavonol myricetin by FLS. DHM also acts as the first substrate leading to the anthocyanidin delphinidin, a purple-colored pigment. Both DHQ and DHK can also be converted into cyanidin and pelargonidin, respectively, the other two main anthocyanidins present in Solanaceae fruits. For the synthesis of these three anthocyanidins, DHM, DHQ and DHK, the enzymes dihydroflavonol 4-reductase (DFR) and subsequently anthocyanidin synthase (ANS) are required instead of flavonol synthase (**Figure 3**; **Supplementary Data S1**). Moreover, both cyanidin and its intermediate leucocyanidin can act as precursors for the formation of another subclass of flavonoids, the flavan-3-ols, mainly represented by catechin and epicatechin. However, the substrate specificity of DFR for dihydromyricetin leads to the occurrence of only the anthocyanidin delphinidin in these three crops. Delphinidin serves as precursor for the formation of anthocyanins, of which delphinidin-3-(*p*-coumaroyl-rutinoside)-5-glucoside is the most predominant in tomato, eggplant and pepper (**Figure 3**). The Solanaceae anthocyanin biosynthetic pathway has been thoroughly analyzed in a recent review, hence, it is not discussed herein (Liu et al., 2018).

Using four eggplant genotypes with contrasting peel pigmentation, their hybrids and a white-fruited line (‘L131’), Lo Scalzo et al. (2021) studied the biochemical and gene expression changes in the peel during fruit development. This work demonstrated that the activation/deactivation of different branches of the phenylpropanoid pathway, occurs during ripening, resulting in a drastic drop of anthocyanins and CGA concentrations as ripening progresses from commercial to physiological maturity, with distinct phenolic compounds accumulating in the eggplant peel instead, mainly naringenin chalcone and naringenin 7-O-glucoside, along with other minor flavanones and flavonols, hence the change to a yellow coloration. Furthermore, the expression profiling of nine key genes (*SmelCHS_ch00, SmelCHS_ch05, SmelCHI_ch10, SmelCHI_ch05, SmelGT_ch01, SmelGT_ch05, SmelGT_ch10, SmelDFR_ch00 and SmelFLS_ch04)*, encoding enzymes involved in different paths of the phenylpropanoid biosynthesis, revealed changes consistent with the compositional results: *CHS* genes are expressed in eggplant fruit peel at all stages of maturity; a drop in the expression of *CHI* genes occurs at commercial and subsequent stages of maturity; a dramatic increase of glycosyltransferase (*GT*) genes expression during the last stages of physiological maturity may indicate their role in the glycosylation of naringenin and naringenin chalcone; and the *FLS* gene was strongly expressed at physiological maturity, as opposed to the *DFR* gene, being a strong candidate underlying the switch between the two branches of the phenylpropanoid pathway (Lo Scalzo et al., 2021).

A similar trend was reported for four pepper cultivars bearing different fruit colors. Liu et al. (2020) performed a targeted metabolome and transcriptome integrative study at two developmental stages. Results confirmed that flavonoid synthesis was developmental-dependent, as higher expression levels of structural genes of the phenylpropanoid pathway were observed at the immature stage for all four genotypes. Comparison of the metabolic profile with expression levels of structural genes, linked a higher expression of *PAL*, *C4H* and *4CL* to a higher accumulation of flavonols, flavones and anthocyanins in pepper fruits. A higher expression of *CHI* resulted in higher accumulation of naringenin, however, as reported by the authors, this was not translated into higher levels of downstream flavonoids due to the low expression of *F3H* and *F3′5′H* genes at green stage. This indicates the key role of these genes in regulating the flux to the lower branches of the phenylpropanoid pathway (Liu et al., 2020).

Naringenin can also act as a precursor for the other two main subclasses of flavonoids: flavones and isoflavones. The latter are mainly found in legumes, nuts and cereals and less relevant in tomato, eggplant and pepper, thus they are not discussed herein. Naringenin can be converted to the flavones apigenin and luteolin through the action of flavone synthases FNS-I and FNS-II, which have been characterized as a 2-oxoglutarate-dependent dioxygenase and a cytochrome P450 monooxygenase from the CYP93 family, respectively. Both enzymes catalyze the addition of a double bond between C-2 and C-3 in the heterocycle of naringenin (Scossa et al., 2019) (**Figure 3**; **Supplementary Data S1**).

It is worth mentioning that despite the structural genes being well conserved across these species, single or multiple mutations, deletions and other alterations to their sequences have been shown to alter significantly the metabolic composition and accumulation pattern in Solanaceae fruits (Chioti et al., 2022).

### 2.2 QTLs and candidate genes controlling phenolics accumulation

Many QTLs encompass the generalist trait of total phenolics content and total antioxidant capacity. Although several classes of compounds may be included in this category, we believe that it is relevant to include them in this review. Because we cannot classify them into specific categories, we kept the generalist designation. **Table 1** summarizes all the QTLs found in the literature associated with phenolic compounds in tomato, eggplant and pepper. For more specific information on the compiled QTLs refer to **Supplementary Data S2**.

**Table 1:** Number of QTLs associated with phenolic acids, flavonoids (other than anthocyanins) and total phenolics content for tomato, eggplant and pepper.

Species	Mapping population	Metabolite(s)	QTLs	Chromosome(s)	Candidate gene(s) (encoding protein)	Reference
**Tomato**	ILs *S. pennellii* (LA0716) x *S. lycopersicum* cv. ‘M82’	Phenolic acids	67	[1-12]	Solyc10g085730, Solyc10g085860, Solyc10g085870, Solyc10g085880, Solyc10g086240 (UGT1, UDP-glycosyltransferase 1), Solyc10g086180(PAL, phenylalanine ammonia lyase), Solyc12g099130, Solyc12g099120, Solyc12g099140, Solyc12g099620, Solyc12g099850 (MYB)	(Alseekh et al., 2015)
		Flavonoids	30	[1-12]	Solyc06g083450 (O-methyltransferase), Solyc06g083470, Solyc06g083480, Solyc06g083490 (3-ketoacyl-acyl carrier protein reductases/tropan reductase), Solyc06g084240 (ent-copalyl diphosphate synthase)	(Alseekh et al., 2015)
		Total phenolics	38	[1-12]	–	(Alseekh et al., 2015)
			9	3, [5-9]	–	(Rousseaux et al., 2005)
			1	7	–	(Sacco et al., 2013)
			1	7	–	(Di Matteo et al., 2010)
			1	7	Solyc10g081260 (MATE, Multidrug resistance protein), Solyc06g005080 (VSP, Vacuolar protein sorting-associated protein 18), Solyc07g056420 and Solyc07g056510 (GST, Glutathione S-transferase), Solyc05g051200 (ERF1, Ethylene Responsive Factor 1)	(Di Matteo et al., 2013)
	BILs and ILs *S. pennellii* (LA0716) x *S. lycopersicum* cv. ‘M82’	Phenolic acids	3*	1, 2, 10, 11	–	(Szymański et al., 2020)
		Flavonoids	8*	1, 5, 9	Solyc05g010310.3 and Solyc05g010320.3 (CHI1, naringenin chalcone isomerase 1)	(Szymański et al., 2020)
	ILs *S. chmielewskii* (LA1840) x *S. lycopersicum* cv. ‘Moneyberg’	Phenolic acids	11	3, 4, 7, 10, 12	Solyc07g005760 (HQT, Hydroxycinnamoyl CoA shikimate/quinate hydroxycinnamoyltransferase)	(Ballester et al., 2016)
		Flavonoids	23	2, [4-9], 12	Solyc05g010320 (CHI1, naringenin chalcone isomerase 1)	(Ballester et al., 2016)
	ILs *S. habrochaites* (LA1777) x *S. lycopersicum* cv. ‘E6203’	Flavonoids	1	5	Solyc05g010320 (CHI1, naringenin chalcone isomerase 1)	(Hanson et al., 2014)
	BC₂F₂ *S. habrochaites* (LA1223) x *S. lycopersicum* ‘TA1166’	Flavonoids	4	2, 3, 5, 11	–	(Ökmen et al., 2011)
		Total phenolics	5	1, 6, 7, 9, 12	–	(Ökmen et al., 2011)
	96 cultivated genotypes (GWAS)	Total phenolics	2	8, 11	Solyc08g082350.2.1 (unknown), Solyc11g010170.1.1 (LanC-like protein2), Solyc11g010200 and Solyc11g010470 (14-3-3), Solyc11g 009100 (ABC-2 Transporter), Solyc11g 010380 (MATE)	(Ruggieri et al., 2014)
	RILs, ILs, Sub-ILs *S. pimpinellifolium* (TO-937) x *S. lycopersicum* cv. ‘Moneymaker’	Total phenolics	10	1, 4, 5, 7, 8, 11, 12	Solyc01g079620 (SlMYB12), Solyc01g079240 (LACS1, LONG CHAIN ACYL SYNTHETASE 1), Solyc01g056340 (DET1, DE-ETIOLATED 1), Solyc04g014370 (PAS2, PASTICCINO2), Solyc05g008250 (GL1, GLABRA1), Solyc08g067260 (FDH, FIDDLEHEAD), Solyc08g067410 (KCS11-like 1)	(Barraj Barraj et al., 2021)
**Eggplant**	F₂ population from ‘305E40’ x ‘67/3’	Phenolic acids	2	4, 6	–	(Toppino et al., 2016)
	ILs *S. melongena ‘*AN-S-26’ × *S. incanum ‘*MM577’	Phenolic acids	1	7	SMEL_007g290860.1 (PAL1, phenylalanine ammonia-lyase 1), SMEL_007g288660.1.01 (peroxidase), SMEL_007g288680.1.01 (peroxidase), SMEL_007g288690.1.01 (peroxidase)	(Rosa-Martínez et al., 2022a)
			4	3, 5, 7, 12	SMEL_003g185030.1 (4CL2, 4-coumarate-CoA ligase 2), SMEL_007g275930.1 (4CL5, 4-coumarate-CoA ligase 5), SMEL_007g290860.1 (PAL6, phenylalanine ammonia-lyase 6)	(Rosa-Martínez et al., 2022b)
		Total phenolics	2	3, 12	SMEL_003g185030.1 (4CL2, 4-coumarate-CoA ligase 2)	(Rosa-Martínez et al., 2022b)
	BC₂ and BC₃ *S. melongena* ‘MEL3’ × *S. elaeagnifolium* ‘ELE2’	Phenolic acids	2	1, 5	–	(Villanueva et al., 2021)
		Total phenolics	2	1, 6	SMEL_006g261420.1.01 (CCR1, cinnamoyl-CoA reductase 1), SMEL_006g261630.1.01 (4CL2, 4-coumarate-CoA ligase 2)	(Villanueva et al., 2021)
	F_6_RIL *S. melongena* ‘305E40’ x *S. melongena* ‘67/3’	Flavonoids	3	7, 10	SMEL_007g288740.1, SMEL_007g288700.1, SMEL_007g288720.1, SMEL_007g288660.1, SMEL_007g288690.1, SMEL_007g288730.1, SMEL_007g288920.1, SMEL_010g353210.1, SMEL_010g352840.1, SMEL_010g352880 (PYL4 abscisic acid receptor), SMEL_010g352930.1, SMEL_010g353090.1, SMEL_010g353110.1, SMEL_010g353170.1 and SMEL_010g353190.1 (kaempferol 3-O-beta-D-sophoroside), SMEL_010g352880 (rutin)	(Sulli et al., 2021)
**Pepper**	F₂ *C. annuum* ‘AC1979’ × *C. chinense* ‘No. 4661’	Phenolic acids	1	11	–	(Wahyuni et al., 2014)
		Flavonoids	32	[1-4], [6-10]	*CaMYB12* (naringenin chalcone, FLS, CHI, CHS)	(Wahyuni et al., 2014)
	NILs, F₂ *C. annuum* ‘Long Sweet’ (P12) × *C. annuum* ‘AC2212’ (P24)	Flavonoids	1	5	Ca05g18430 (*CaMYB12-like*, MYB-like DNA-binding domain protein)	(Wu et al., 2022)

* indicates mQTL hotspots.

Information regarding the mapping population used, chromosome, candidate genes within the QTLs regions and reference is also provided.

*Tomato*

Several interspecific tomato mapping populations have been used to study the genetics of phenolics, yielding an array of QTLs and candidate genes. The most studied mapping population is probably the one of *S. pennellii* (LA0716) in the genetic background of *S. lycopersicum* cv. ‘M82’ (LA3475) (Eshed and Zamir, 1995). Using this population, Alseekh et al. (2015) identified 67 and 30 robust (P < 0.01) metabolic QTLs (mQTLs) controlling hydroxycinnamates and flavonoids accumulation in the fruit pericarp, respectively (**Table 1**). The heritability of a pool of 23 UPLC-FTMS-identified hydroxycinnamates was high for 11 (e.g., homovanillic acid hexose II, caffeoyl-hexose II), intermediate for four, and low for eight. Likewise, the identified flavonoids showed high heritability, namely naringenin chalcone, with only two displaying intermediate and one low heritability. Furthermore, hydroxycinnamate QTLs showed a prevalent dominant negative mode of inheritance, with heterozygote lines showing lower levels of many compounds (e.g., coumaric acid hexose I and II, homovanillic acid hexose II), whereas flavonoids were reported to be split between dominant and additive negative QTLs. Few traits showed recessive mode of inheritance (Alseekh et al., 2015). The high frequency of negative mQTLs, as pointed out by the authors, suggests transcriptional regulation. In this way, 17 different families of transcription factors (TFs), detected by qRT-PCR, correlated to the expression of mQTLs, including MYB and MADS families (**Table 1**; **Supplementary Data S2**). The analysis of sequence polymorphisms of TF-encoding genes showed several variations between wild and cultivated sequences with potential impact on translation, stability, expression level, and tissue specificity (Alseekh et al., 2015). The regulation of the phenylpropanoid pathway is discussed in more detail in section 2.3.

Furthermore, Alseekh et al. (2015) identified 38 robust metabolic mQTLs controlling total phenolics content in the fruit pericarp scattered across the 12 chromosomes (**Table 1**; **Supplementary Data S2**). Likewise, Rousseaux et al. (2005) reported nine QTLs (*phe*3-2, *phe*3-3, *phe*3-4, *phe*5-4, *phe*6-2, *phe*7-2, *phe*7-4, *phe*8-2 and *phe*9-1) controlling total phenolics content in this population, although only one was found in both years of the experiment (*phe*6-2). The stable QTL increased phenolics concentration by 40%, on average, compared to ‘M82’. Both *phe*6-2 and *phe*7-4 showed a positive effect, whereas the other QTLs (often carrying the *S. pennellii* allele) had a negative impact on phenolics levels. The authors suggested that a complex regulatory system was expected based on the F_1_ intermediate values (Rousseaux et al., 2005) (**Table 1**; **Supplementary Data S2**). Di Matteo et al. (2010) and Sacco et al. (2013) explored the expression, location and inheritance model of the QTL identified in IL7-3 (LA4066), responsible for higher levels of phenolics, located at 39-64 cM in chromosome 7, and confirmed its recessive mode of inheritance. Finally, Di Matteo et al. (2013), using transcriptomics and TILLING, proposed that the higher phenolics content of IL7-3 is due to a more efficient compartmentalization in the vacuoles of these metabolites (specially flavonoids), as a result of higher expression of *MATE* (Solyc10g081260), *VSP* (Solyc06g005080), and *GST* (Solyc07g056420 and Solyc07g056510) genes, ultimately regulated by *ERF1* (Solyc05g051200) located at chromosome 5 (**Table 1**; **Supplementary Data S2**).

More recently, a population of backcross inbred lines (BILs) was developed from the *S. pennellii* ILs to generate a many-fold higher mapping resolution (Ofner et al., 2016). A total of 504 of these BILs, along with 76 ILs with *S. pennellii* introgressions were evaluated by Szymański et al. (2020) using a multi-omics approach, which resulted in the identification of several flavonoid-related mQTLs, as well as eQTLs associated with the phenylpropanoid metabolism. As an outcome worth exploring, the authors provided an online repository of the large dataset generated (https://szymanskilab.shinyapps.io/kilbil/) to make it easily accessible. Most notably, a large mQTL in chromosome 5 (bin 424) was found to be a hotspot for metabolic changes of several metabolites, including at least 22 flavonoids that were downregulated at the red fruit stage, as well as rutin and four kaempferol glycosides that were upregulated. We summarized in **Table 1** the most relevant mQTL hotspots for phenolic acids, flavonoids, and derivatives, which were highlighted by the authors (Szymański et al., 2020) (**Table 1**; **Supplementary Data S2**). Furthermore, this study revealed the key role of specific flavonoids in fruit response against *Botrytis cinerea*. In this way, the authors reported the association of a higher accumulation of naringenin-7-O-glucoside with susceptibility, while a higher accumulation of its aglycone naringenin was associated with the resistant phenotype. (Szymański et al., 2020).

An IL population derived from a cross between *S. chmielewskii* (LA1840) and *S. lycopersicum* cv. ‘Moneyberg’ has also provided important insights into the phenolic acids and flavonoids accumulation in tomato fruits. Through LC-PDA-QTOF-MS coupled with physical mapping of the introgressions, Ballester et al. (2016) identified 126 different compounds from different classes, including phenolic acids and flavonoids, 56 of which were significantly increased/decreased compared to the cultivated parent. Thus, an introgression in chromosome 7 (IL7d) was linked to significant increases of di- and tri-caffeoylquinic acid and chlorogenic acid. Interestingly, within this region is located the gene *HQT*, responsible for trans-esterification of caffeoyl-CoA with quinic acid in order to form CGA. IL4d was linked to an increase of tri-caffeoylquinic acid, chlorogenic acid and 4-caffeoylquinic acid. Contrastingly, an introgression in chromosome 12 (IL12d) showed almost a three-fold decrease in di- and tri-caffeoylquinic acid compared to the cultivated accession (Ballester et al., 2016). Likewise, IL5b was found responsible for the significant increases of kaempferol and quercetin glycosides in the fruit peel. This region proved to harbor 511 genes, although only 17 were upregulated three-fold or higher, one encoding CHI (Solyc05g010320), which is responsible for directing the flux of the pathway towards the synthesis of flavonol glycosides (Muir et al., 2001). Similarly, IL4d showed potential to increase kaempferol and quercetin glycosides, although in a more modest way (between 3- and 5-fold compared to controls). Other introgressions showed a positive effect on one or two metabolites, like IL2b for quercetin feruloyl deoxyhexose-dihexose or IL6e for quercetin coumaroyl hexose-deoxyhexose-hexoside. Contrarily ILs 8a, 9d and 12d showed a significant negative impact on the concentration of quercetin and naringenin glycosides (Ballester et al., 2016) (**Table 1**; **Supplementary Data S2**).

Seven ILs derived from a cross between *S. habrochaites* (LA1777) and *S. lycopersicum* cv. ‘E6203’ (Monforte and Tanksley, 2000) were selected to be further analyzed regarding their chemical profiles (Hanson et al., 2014). Introgression line LA3984, encompassing a small fragment of *S. habrochaites* in chromosome 5, showed high levels of rutin in ripe fruits. The QTL proved to be harboring 38 genes, including the annotated chalcone-flavonone isomerase gene (Solyc05g010320), which was proposed as the gene responsible for high rutin levels in the *S. chmielewskii* IL population (Ballester et al., 2016). In addition, a second chalcone-flavonone isomerase gene was located upstream the QTL (**Table 1**; **Supplementary Data S2**). The introgression of this *S. habrochaites* QTL increased the expression of chalcone isomerase by restoring the flavonol synthesis pathway, resulting in elevated rutin content (Hanson et al., 2014).

Using the BC_2_F_2_ population from *S. habrochaites* (LA1223) × *S. lycopersicum* ‘TA1166’, Ökmen et al. (2011) reported five QTLs, scattered across chromosomes 1, 6, 7, 9 and 12, controlling the total phenolics fraction of tomato fruits (**Table 1**; **Supplementary Data S2**). All QTLs showed a positive effect and two coincided with Rousseaux et al. (2005) findings, at chromosome 7 and 9. For these QTLs, the wild allele increased the phenolics content between 8 and 17%. The QTL located at chromosome 7 showed the biggest increase in phenolics content (Ökmen et al., 2011). Moreover, authors reported four QTLs controlling flavonoid accumulation in tomato fruits, one with a positive effect, located at chromosome 11, and three with negative effect, located at chromosomes 2, 3 and 5. Despite the negative effect of most of the wild alleles, the QTL located at chromosome 11 increased the flavonoid content by 24% (**Table 1**; **Supplementary Data S2**).

A GWAS was carried out by Ruggieri et al. (2014) to detect associations between SNPs and fruit nutritional and apparent quality traits in a collection of 96 cultivated tomato genotypes, using the SNP-based SolCAP array. Two polymorphic markers were significantly associated with total phenolics content, located at chromosomes 8 (solcap_snp_sl_100367) and 11 (solcap_snp_sl_34253) (**Table 1**; **Supplementary Data S2**). In both cases, the minor alleles had a positive effect on the trait. The first marker was associated with gene Solyc08g082350.2.1, encoding for a protein of unknown function, while the second was associated with gene Solyc11g010170.1.1, which encodes a LanC-like protein 2, which is involved in the modification and transport of peptides in bacteria, although no clear role has been attributed in plants. In addition, four candidate genes were in linkage-disequilibrium with the marker at the beginning of chromosome 11, two *14-3-3* (Solyc11g010200 and Solyc11g010470), one *ABC-2 Transporter* (Solyc11g 009100) and one *MATE* (Solyc11g 010380) (Ruggieri et al., 2014). Interestingly, the *MATE* had been reported to be involved in vacuolar compartmentalization of phenolic compounds by Di Matteo et al. (2013) (**Table 1**; **Supplementary Data S2**).

More recently, the study of RILs, ILs and SubILs derived from a cross between *S. pimpinellifolium* (TO-937) and *S. lycopersicum* cv. ‘Moneymaker’ identified nine QTLs (*ph*1.1, *ph*4.1, *ph*5.1, *ph*5.2, *ph*7.1, *ph*7.2, *ph*8.1, *ph*12.1 and *ph*12.2) involved in the accumulation of cuticle phenolics across six different chromosomes (Barraj Barraj et al., 2021) (**Table 1**; **Supplementary Data S2**). Two wild alleles, *ph*4.1 and *ph*12.1, were linked to an increase of 20 and 30% in ripe fruits, respectively, while alleles located at chromosome 5 showed increased phenolics levels at immature and mature green stages. The other five QTLs, carrying the cultivated allele, showed a negative effect on the accumulation of phenolics in the fruit peel. The cultivated QTL *ph*12.2 had a particularly negative impact, leading to a 40% decrease of phenolics. The QTL in chromosome 1 was linked to *SlMYB12* (Solyc01g079620), *LONG CHAIN ACYL SYNTHETASE 1* (*LACS*1; Solyc01g079240) and *DE-ETIOLATED1* (*DET1*; Solyc01g056340) genes, although closer to the first one. The QTL *ph*4.1 co-located with *PASTICCINO2* (*PAS2*; Solyc04g014370), *ph*5.1 with *GLABRA1* (*GL1*; Solyc05g008250), and *ph*8.1 with *FIDDLEHEAD* (*FDH*; Solyc08g067260) and *KCS11-like1* (Solyc08g067410). Many of these genes have roles in cuticle deposition, but could be also involved in the phenolics transport to the cuticle, as the expression analysis seemed to suggest for *FDH* (Barraj Barraj et al., 2021) (**Table 1**; **Supplementary Data S2**). Furthermore, phenolics gene expression seems to be regulated by a complex combination of additive and epistatic interactions, with tight links to the cuticle wax and polysaccharides synthesis, as reported by España et al. (2014) and Heredia et al. (2015). An epistatic relationship between a region in chromosome 11 and the QTL found in chromosome 12 (*ph*12.1) was detected and, although the region on chromosome 11 had no significant effect on the phenolics content, it increased the effect of *ph*12.1 (Barraj Barraj et al., 2021).

Tomato mutants, carrying natural or induced mutations, may also represent a source of valuable alleles regarding metabolic regulation of different pathways (Bovy et al., 2010; Chattopadhyay et al., 2021). In that regard, *high pigment* (*hp*) tomatoes comprise a particularly interesting source of variability since their phenotype is the result of exaggerated accumulation of many antioxidants and photoprotective metabolites, including flavonoids, in their fruits (Levin et al., 2006; Azari et al., 2010; Chattopadhyay et al., 2021). The different tomato *hp* mutants contain mutations in either the *DET1* gene (*hp-2*, *hp-2^j^*, and *hp-2^dg^*), a negative regulator of photomorphogenesis, or the *UV DAMAGED DNA BINDING* protein 1 (*DDB1*) gene (*hp-1* and *hp-1^w^*) which interacts with the *DET1* gene (Levin et al., 2006; Azari et al., 2010). Several studies reported the metabolic profiles of the different *hp* mutants and showed that these mutations not only led to the accumulation of high levels of flavonoids, but also to increased levels of other bioactive compounds, such as carotenoids (Bino et al., 2005; Levin et al., 2006; Long et al., 2006; Rutley et al., 2021). The *hp* mutations have been extensively introgressed into elite cultivars, mainly to increase the lycopene content in processing tomatoes (Levin et al., 2006). Likewise, different combinations of *hp* mutations have been stacked in the same genetic background to shed light on their genetic control and their impact on metabolite content. The homozygote double-mutants carrying *Anthocyanin fruit* (*Aft*) with *hp-1^w^* and *Aft* with *hp-2^j^* showed a significant increase in the accumulation of both anthocyanins and rutin compared to the single-mutation lines (van Tuinen et al., 2006). In addition, the stacking of *Aft* with *hp-1* showed promising results by synergistically increasing the production of delphinidin-, petunidin- and malvidin-type anthocyanins and quercetin- and kaempferol-type flavonols in the fruit (Sapir et al., 2008).

*Eggplant*

Being the most abundant phenolic acid in eggplant fruits, studies of the phenolic profile have naturally focused on CGA. Although few QTL approaches have been carried out for deciphering the genetic basis of CGA biosynthesis in eggplant, the development of a growing number of intra and interspecific mapping populations is leading to an escalation of these studies. A F_2_ population derived from an intraspecific crossing (‘305E40’ × ‘67/3’) was used to identify two stable QTLs for CGA content on linkage groups E04 (*CGAE04*) and E06 (*CGAE06*) (Toppino et al., 2016). In both cases the positive allele came from the ‘305E40’ parent. No candidate genes pointing at the phenylpropanoid pathway could be identified within the QTLs’ regions. However, according to the authors, *CGAE04* and *CGAE06* could be related to quinic acid, since they were syntenic to two tomato ILs containing QTLs for that moiety of CGA (Toppino et al., 2016) (**Table 1**; **Supplementary Data S2**). A stable QTL associated with a reduction of CGA content was identified in a recent work (Rosa-Martínez et al., 2022a) using the first developed set of interspecific eggplant ILs with genome fragments of its wild relative *S. incanum* (Gramazio et al., 2017). The QTL (*cga7*) was located at the end of chromosome 7 (129-135 Mb), where a potential candidate gene (SMEL_007g290860.1), orthologous to the tomato Solyc09g007920, which encodes for phenylalanine ammonia-lyase 1 (*Sl*PAL1), was identified by the authors (**Table 1**; **Supplementary Data S2**). In a different trial, under two nitrogen (N) regimes, four QTLs for decreased CGA content, on chromosomes 3, 5, 7 and 12, as well as two QTLs negatively controlling total phenolics content, on chromosomes 3 and 12, were identified (**Table 1**; **Supplementary Data S2**) (Rosa-Martínez et al., 2022b). Most of these QTLs were only detected under high-N conditions, whereas the QTL for CGA on chromosome 3 was identified under the two N treatments (*cga3.HN* and *cga3.LN*). However, the introgressed fragment large size hampered the identification of underlying candidate genes and, therefore, it will require the use of strategies such as obtaining sub-ILs, to narrow it down (Rosa-Martínez et al., 2022b). Finally, the evaluation of a set of BC_2_ and BC_3_, derived from the cross of *S. melongena* ‘MEL3’ and its distant wild relative *S. elaeagnifolium* ‘ELE2’ (García-Fortea et al., 2019), under N-restrictive conditions, led to the identification of a QTL increasing CGA content on chromosome 5 (3.9–4.5 Mb) (Villanueva et al., 2021). Interestingly, a distinct pattern of phenolic acid accumulation, associated with a larger array of hydroxycinnamate conjugates other than CGA was detected in this population. The authors were able to identify a QTL associated with the unusual phenolic profile of *S. elaeagnifolium* on chromosome 1, as well as two QTLs associated with a greater total phenolic HPLC-peaks area and a lower CGA HPLC-peak area, located on chromosomes 6 and 1, respectively (Villanueva et al., 2021) (**Table 1**; **Supplementary Data S2**).

Studies about the flavonoid accumulation, other than anthocyanins, in eggplant are scarce, due to the fact that their concentration is negligible compared to CGA and anthocyanins at commercial maturity (Lo Scalzo et al., 2021). A recent metabolic study of a F_6_ recombinant inbred line (RIL) population (Sulli et al., 2021) derived from the eggplant lines ‘305E40’ and ‘67/3’, identified one mQTL on chromosome 10 associated with the level of rutin (P-RUT.10.1). Two other mQTLs were associated with the level of the flavonol glycoside kaempferol 3-*O*-beta-D-sophoroside. One of them was also located on chromosome 10 (P-KSOPH.10.1) near P-RUT.10.1, while the other was located on chromosome 7 (P-KSOPH.7.1). Several candidate genes for these QTLs were also suggested by the authors, encompassing a PYL4 abscisic acid receptor (SMEL_010g352880), which is known to positively regulate flavonoid/anthocyanin biosynthesis in tomato (Diretto et al., 2020), peroxidase-encoding genes and other transcription factors (Sulli et al., 2021) (**Table 1**; **Supplementary Data S2**).

*Pepper*

Only a few studies aimed at identification of QTLs associated with phenolic compounds were found for pepper at the time of writing. In the first work, authors used a genetic linkage map constructed from an interspecific F_2_ population derived from the cross between *C. annuum* AC1979 and *C. chinense* No. 4661 to locate QTLs and genes (Wahyuni et al., 2014). From the comprehensive untargeted metabolic profiling, which led to the identification of more than 500 semi-polar compounds, the authors employed both the QTL and candidate gene approaches to identify one mQTL for the phenolic acid derivative ferulic acid-hexose located in the linkage group P11 (Wahyuni et al., 2014) (**Table 1**; **Supplementary Data S2**). In terms of flavonoids, this study identified 12 mQTLs for different flavonoid glycosides encompassing quercetin, apigenin, luteolin, flavanone derivatives, and naringenin chalcone, scattered over the linkage groups P02, P06, P07, P09 and P10. Moreover, 15 eQTLs for flavonoid pathway genes were identified: (i) eQTLs for *CHS-1*, *CHS-2*, *CHI-2* and *FLS* co-localized on P01 and overlapped with a mQTL for naringenin chalcone, eQTLs for *CHS-1*, *CHS-2*, *CHI-1*, *CHI-2* and *FS-2* co-localized with a naringenin chalcone mQTL on P09, (iii) eQTLs for *F3’H-1* and *F3’H-4*, and *F3H* were detected on P03, P04 and P08, respectively (Wahyuni et al., 2014) (**Table 1**; **Supplementary Data S2**). In a study aimed at identifying QTLs associated with *α*-glucosidase inhibitory activity of flavonoids and other compounds present in pepper leaves and fruits (Park et al., 2020), the authors found a QTL on chromosome 9, which co-located with the cluster of mQTLs and eQTLs identified by Wahyuni et al. (2014). In a recent work, Wu et al. (2022) used a F_2_ population, resulting from the cross between the flavonoid-contrasting *C. annuum* accessions Long Sweet (P12) and AC2212 (P24), to identify a major QTL (*fc5.1*) controlling the accumulation of chalcones, flavonols and flavones located at 228 Mbp on pepper chromosome 5. Further analysis of this region, in a NILs (Near Isogenic Lines) population derived from the original F_2_ population, identified three *CHS* (Ca5g17030, Ca5g17040 and Ca5g17060) and a homolog to the tomato TF gene *SlMYB12*, *CaMYB12-like* (Ca05g18430). RNA-seq later confirmed the up-regulation of *CaMYB12-like* in NILs carrying the high-flavonoid P12 allele along with 17 other genes involved in the flavonoid pathway. Since this network of genes is scattered across several chromosomes, *CaMYB12*-like must be *trans*-regulating the entire pathway. Virus-induced gene silencing (VIGS) confirmed that *CaMYB12-like*-silenced plants had a significant decrease of flavonoid content and structural flavonoid pathway genes expression levels, compared to mock-silenced plants (Wu et al., 2022).

### 2.3 Regulation of phenylpropanoid biosynthesis pathway

Although the phenylpropanoid biosynthetic pathway is conserved across these species, the accumulation profile of metabolites in tomato, eggplant and pepper differs significantly (Rosa-Martínez et al., 2021). Moreover, studies have proven that phenylpropanoids accumulate in a tissue-specific and developmental-dependent ways (Calumpang et al., 2020; Liu et al., 2020; Lo Scalzo et al., 2021). This implies that, in addition to the structural genes, the pathway is tightly controlled by regulatory complexes. Transcription factors (TFs) of the R2R3-type MYB, Zinc Finger, bHLH, MADS, WRKY and WD40 families have been reported to regulate the phenylpropanoid metabolism in plants, including the Solanaceae (Tohge et al., 2017; Zhu et al., 2018; Sacco et al., 2019; Liu et al., 2020). Among these, the R2R3-type MYB TFs are the most relevant group in tomato, eggplant and pepper.

The role of *MYB12* in the regulation of phenylpropanoid metabolism has been thoroughly characterized in tomato. The study of the metabolomic and molecular profiles of pink-colored fruits of two sets of ILs with wild introgressions from *S. pennellii* (Adato et al., 2009) and *S. chmielewskii* (Ballester et al., 2010) linked *SlMYB12* to the genomic region harboring the *y* mutation on chromosome 1, and to the regulation of naringenin chalcone accumulation in the fruit peel. This was demonstrated by downregulation/upregulation (Adato et al., 2009), gene silencing (Ballester et al., 2010), overexpression (Wang et al., 2018) and by studying mutations in its sequence (Fernandez-Moreno et al., 2016; Jung et al., 2017). These determined that *SlMYB12* controls the expression of the structural genes *SlPAL*, *Sl4CL*, *SlCHS*, *SlCHI*, *SlF3H*, *SlF3’H* and *SlFLS*, being the main activator of the flavonol biosynthetic branch. In addition, Zhang et al. (2015) demonstrated that *AtMYB12*, *SlMYB12*’s homolog of *Arabidopsis*, is not only able to bind to specific phenylpropanoid structural genes, but also to genes associated with the primary metabolism, such as 3-deoxy-D-arabino-heptulosonate 7-phosphate synthase (*DAHPS*) and plastidial enolase (*ENO*), and thus exerting its activity at several metabolic points. This complex regulatory action of *SlMYB12* was also pointed out by Fernandez-Moreno et al. (2016) through transcriptomics and by Zhu et al. (2018). In this way, besides increasing the demand for flavonoid synthesis, *MYB12* would increase the flow of carbon entering the shikimate pathway and the formation of aromatic amino acids that serve as substrates for the synthesis of phenolic compounds (Zhang et al., 2015). Other MYB-type TFs have been reported to act as regulators at different levels of both primary and phenylpropanoid metabolism in tomato. Wu et al. (2020) reported a significant impact of *SlMYB72* on several vital metabolic pathways, including the phenylpropanoid pathway. Downregulation of *SlMYB72* led to increased levels of total flavonoids, quercetin, kaempferol, gallic acid and chlorogenic acid, whereas *SlMYB72*-upregulated plants showed no significant differences compared to their azygous controls. *SlMYB72* was reported as a significant player in the regulation of the phenylpropanoid and flavonoid pathways, since it directly influenced the expression of *4CL*, *CHS1* and *CHS2* genes (Wu et al., 2020).

In eggplant, efforts in this area so far resulted in the identification of more than 100 R2R3-MYB TFs (Li et al., 2021), categorized into 20 subgroups (SGs), of which four (SG4 to SG7) were reported to modulate the phenylpropanoid pathway, mainly anthocyanin and flavonol biosynthesis. Within these SGs, ten genes were identified by the authors. *SmMYB1* (SG6), was isolated from an occidental eggplant variety with the purpose of characterizing its expression level in different tissues and its regulation mechanism (Docimo et al., 2016). *SmMYB1* is orthologous to *SlANT1* and the locus *A* in *C. annuum*, widely characterized in tomato and pepper, respectively, as main regulators of anthocyanin synthesis. The authors found that *SmMYB1* was responsible for differentially regulating both the chlorogenic acid and anthocyanin biosynthesis in eggplant. Through *N. benthamiana* transient expression of *SmMYB1* and a C-terminal truncated form of the protein, the authors demonstrated the essential role of the C-terminal region in anthocyanin biosynthesis, while its deletion does not limit the capability of *SmMYB1* to regulate chlorogenic acid accumulation. These results pointed out that TFs involved in the phenylpropanoid pathway might contain several action domains that can bind to different substrates and exert their regulatory action at different points along the pathway (Docimo et al., 2016).

In pepper, using sequence homology with *SlMYB12*, Wahyuni et al. (2014) located a candidate gene for *CaMYB12* in chromosome 1 in the F_2_ population derived from *C. annuum* × *C. chinense* genetic linkage map. In addition, through metabolic and gene expression analyses in ripe fruits of this population, the authors identified a mQTL for naringenin chalcone and eQTLs associated with *CaMYB12* and other genes encoding specific enzymes in the flavonoid pathway (*CaCHS-1*, *CaCHS-2*, *CaCHI-1* and *CaFLS*), which colocalized at the *CaMYB12* locus on chromosome 1 (Wahyuni et al., 2014). This suggests that a similar regulatory action of *MYB12* transcription factor may control the phenylpropanoid metabolism in pepper and tomato.

In a recent publication, Arce-Rodríguez et al. (2021) performed *in silico* genome-wide analyses of the MYB family in *C. annuum* to identify 235 non-redundant MYB proteins. Coupled with RNA-seq and RT-qPCR assays, the authors reported several *CaMYB*s putatively regulating phenylpropanoid biosynthesis in pepper. Thus, *CaMYB32*, *CaMYB33* and *CaMYB93* expression patterns were shown to be co-expressing with *PAL*. Of these, *CaMYB93* expression correlated with the *PAL* expression profile throughout fruit development, whereas *CaMYB32* and *CaMYB33* transcription factors showed to be highly correlated with flower and early fruit development expression. Moreover, the phylogenetic analysis of *CaMYB*s sequences grouped them in 46 clades (C1–C46) indicating both gene conservation and specialization, by grouping some *CaMYB*s with *A*. *thaliana* and tomato MYBs and some in *Capsicum*-only groups, respectively. Hence, *CaMYB97*, *CaMYB98*, *CaMYB99* and *CaMYB100*, which grouped together with *AtMYB12* and *SlMYB12*, could be implicated in the regulation of flavonoid biosynthesis in pepper (Arce-Rodríguez et al., 2021).

Islam et al. (2021) followed a similar approach to identify MYB TFs in the *C*. *chinense*, *C*. *baccatum* and *C*. *annuum* genomes, yielding 251, 240 and 245 unique TFs genes, respectively. Based on expression patterns during fruit developmental stages, researchers identified several *C*. *chinense* and *C*. *annuum* MYB genes co-expressing with *CHS* and *DFR*, two structural genes of the flavonoid pathway, suggesting that *CcMYB16*, *CcMYB28*, *CcMYB100*, *Cc*A, *CcDIV4*, *CcMYB46*, and *CcMYB74* may be regulating anthocyanin/flavonoid biosynthesis in *Capsicum* fruits. In addition, *CcPHR8* and *CaHHO1*, two TFs present within a reported QTL controlling fruit pungency, were also observed to be co-expressing with genes *C4H* and *DFR*, while *CcMYB48* co-expressed with *PAL*, indicating potential involvement in the phenylpropanoid pathway. Through phylogeny the authors confirmed Arce-Rodríguez et al. (2021) results regarding the clustering of MYBs 98, 99 and 100 with *AtMYB12* and thus having a potential role on flavonol glycoside accumulation. Moreover, *CcMYB115* and *CcA* clustered with *AtMYB75* and *AtMYB90* which have been related to anthocyanin and phenylpropanoid biosynthesis, respectively (Islam et al., 2021).

## 3 Breeding strategies for improving phenolics content in tomato, eggplant and pepper

The current knowledge on the genetics of the phenylpropanoid pathway of tomato, eggplant and pepper, including QTLs, structural and regulatory genes, as well as the elite germplasm, provides enough tools to improve phenolics levels, and thus the health-promoting function, of three of the most consumed vegetables in the world. Several works have proved the effectiveness of combining molecular tools and trait variability through conventional and genetic engineering strategies.

### 3.1 Conventional breeding

Great diversity has been found among the varieties of tomato, eggplant and pepper with respect to phenolic acids and flavonoid concentration in fruit. For instance, in tomato, rutin values ranged from 2 to 231 mg kg^-1^ of fresh weight (FW) in a collection of commercial materials and between 22 to 80 mg kg^-1^ FW in a collection of traditional varieties (Bovy et al., 2010; Rosa-Martínez et al., 2021). Stommel and Whitaker (2003) found differences of nearly 20-fold for total hydroxycinnamic acid content in a collection of 101 cultivated eggplant accessions from the USDA-ARS core collection. Mori et al. (2013) also found varietal variations in CGA content between 0.1 and 2.5 mg g^-1^ FW among 34 eggplant cultivars and lines with diverse growth habits, fruit shapes, sizes, and colors. Huge variation has been reported for phenolic acids and flavonoids in pepper varieties, such as vanillic acid (3.1–13.3 mg kg^-1^ FW), ferulic acid (2.2–12.5 mg kg^-1^ FW), quercetin (3.3–783 mg kg^-1^ FW) or luteolin (0.2–103 mg kg^-1^ FW) (Lemos et al., 2019). In addition, many elite materials come from mapping populations. These are incredibly useful genetic resources as they carry favorable alleles for the accumulation of metabolites, usually introgressed from wild accessions, such as *S. pennellii* (LA0716), *S. chmielewskii* (LA1840), *S. habrochaites* (LA1777), *S. pimpinellifolium* (TO-937), S. *incanum* (MM577) and *S. elaeagnifolium* (ELE2), into a cultivated genetic background, that can be readily incorporated into breeding programs (Prohens et al., 2017).

Therefore, the main aspects of conventional breeding should be cataloguing, selecting and hybridizing these elite materials. In addition, to successfully employ the available genetic resources, the identification of QTLs and the underlying genes is of paramount importance. The application of fine-mapping tools and the development of functional markers could have an unprecedent impact in the development of high-phenolic varieties without the negative impact of linkage drag (Salgotra and Neal Stewart, 2020).

As outlined before, the flavonoid pathway is disabled in the tomato fruit flesh of the cultivated germplasm, rendering it impossible to restore just by cross-pollinating the best materials in the primary genepool (Willits et al., 2005). Fortunately, within the wild genepool, there are materials that could address both the restoration of the flavonoid pathway in the fruit flesh and the increase of levels of flavonols in the fruit peel (Tohge et al., 2020). The aforementioned works of Willits et al. (2005); Hanson et al. (2014) and Ballester et al. (2016) are proof of that.

Marker-assisted selection and pyramiding of genes and QTLs have been proven to be effective strategies for improving several traits simultaneously or a single quantitative trait with polygenic control (Sacco et al., 2013; Rigano et al., 2014; Rigano et al., 2016). Numerous QTLs and genes involved in the phenylpropanoid pathway are scattered throughout the genome. The high recombination frequencies among these would make it easier to combine several favorable alleles in a single line. In tomato, this has been successfully used to increase phenolics content (Sacco et al., 2013; Rigano et al., 2014; Rigano et al., 2016). It has also served to demonstrate that the introgressed alleles of *S. pennellii* had a synergistic effect over phenolic acids concentration, mainly gallic and ferulic acids, while being safe for human consumption (Rigano et al., 2014). Also, the combination of transcriptomics, metabolomics and genomics of two pyramided genotypes, obtained by crossing *S. pennellii* IL12-4 and IL7-3, provided insights into the function of structural and regulatory genes involved in the branching of the phenylpropanoid pathway (Rigano et al., 2016). It demonstrated the central role of one 4-coumarate-CoA ligase in the redirection of the pathway towards the synthesis of cinnamic acids or flavonoids, as well as the relevancy of the regulatory action of TFs (Rigano et al., 2016).

In eggplant, higher levels of antioxidant activity, total phenolics and chlorogenic acid have been reported in the wild relative *S. incanum* compared to *S. melongena* (Stommel and Whitaker, 2003; Kaur et al., 2014). Prohens et al. (2013) evaluated the BC_1_ population derived from their crossing for total phenolics and hydroxycinnamic acids content and found a number of plants presenting a good combination of phenolic acids content and fruit weight or flesh browning. Therefore, these plants could constitute a powerful resource to be introduced into breeding pipelines.

Finally, in pepper, the locus *fc5.1*, carrying a high-flavonoid allele, was introgressed into the commercial cultivar backgrounds of Yolo Wonder and Sweet Banana. The resulting BC_1_S_1_ plants revealed an additive effect of this allele on the flavonoid content, showing significant increases of chalcones, flavones and flavonols (Wu et al., 2022).

### 3.2 Genetic engineering

Genetic engineering tools have enabled a great deal of important discoveries regarding the tomato phenylpropanoid pathway. In several cases, it led to outstanding results, unlikely to be achieved through conventional techniques (Schijlen et al., 2006; Bovy et al., 2007). However, the commercialization of genetically modified fruits is not yet legally accepted in some parts of the world, such as Europe. Even the precise gene-editing technology CRISPR-Cas9 has been considered a genetically-modifying technique and is thus under the same limitations (Biswas et al., 2021).

Despite those limitations, CRISPR/Cas9 is becoming the most widespread gene editing technology. Hence, it has been successfully established in Solanaceous crops and has proven its value in applied research directed at understanding the genotype-phenotype relationship. While being primarily reported for tomato, its application in eggplant and pepper is growing (Van Eck, 2018). Genome-editing technologies are enabling researchers to reproduce directly in elite lines the allelic variations responsible for improvement of the traits of interest (Rothan et al., 2019), saving years during the breeding process and avoiding linkage drag of undesired genes/alleles (Deng et al., 2018; Zhu et al., 2018). Some examples of its application in Solanaceae encompass the rewiring of the GABA metabolic pathway in tomato (Li et al., 2018b), the generation of knock-out mutants with reduced PPO activity and browning of eggplant’s fruit flesh (Maioli et al., 2020), and the enhancement of resistance against anthracnose in pepper (Mishra et al., 2021). CRISPR/Cas9 has been successfully applied to edit the phenylpropanoid pathway in tomato. Concretely, researchers generated pink-fruited tomatoes *via* knockout of *SlMYB12* in the genetic background of different red-fruited lines. This led to transparent peels and reduced transcription of *CHS* (Deng et al., 2018; Zhu et al., 2018). By doing so, the authors showed that it is an efficient way to engineer fruit color and the metabolic pathway in tomato without jeopardizing other traits.

Breeding for enhanced flavonoid content in tomato can be approached in two ways: increasing the amount stored in the peel or activating the flavonoid pathway in the fruit flesh. Since the flavonols quercetin and kaempferol have higher bioactive properties, the main goal should be to increase their content. Through transgenesis, Muir et al. (2001) expressed in tomato plants the *Petunia hybrida CHI*, which led to an increase of peel quercetin and kaempferol glycosides content up to 78-fold. Despite that, flesh flavonoid content remained unaltered. On the other hand, the simultaneous ectopic expression of four *P. hybrida* genes (*CHI, CHS, F3H* and *FLS*) was able to produce high flavonol content in the peel and modest levels in the flesh (Colliver et al., 2002; Verhoeyen et al., 2002). Further experiments with transformed tomato plants carrying isolated or different combinations of *P. hybrida* genes led to increased accumulation of naringenin glycosides when *PhCHS* was ectopically expressed, and to those glycosides being converted to flavonol glycosides when *PhCHS* and *PhFLS* were stacked in the same background (Colliver et al., 2002; Verhoeyen et al., 2002). In another approach to restore the flavonoid pathway in the tomato flesh, Bovy et al. (2002) developed transgenic plants expressing the maize TFs *Lc* (*Leaf color*; MYC-type) and *C1* (*colorless-1*; MYB-type) genes, simultaneously, under the fruit-specific E8 promotor control. Authors reported increases of up to 60-fold of kaempferol glycosides in the fruit flesh (Bovy et al., 2002).

Modulation of regulatory genes has been a primary target towards improving the fruit content in flavonoids. Davuluri et al. (2005) used RNAi to suppress the accumulation of *DET1* transcripts in transgenic fruits to increase fruit bioactive compounds content while avoiding the negative impacts on plant development. This resulted in significant increases of naringenin chalcone, flavonol glycosides, chlorogenic acid, and carotenoids with no significant impact on other quality and growth parameters. Lim and Li (2017a) proposed ectopic co-expression of two TFs, *Delila* (bHLH-type) and *Rosea1* (MYB-type) (*Del*/*Ros1*) from *Antirrhinum majus*, in combination with the *Allium cepa CHI* gene in order to produce transgenic ‘Rubion’ tomatoes with high levels of both anthocyanins and flavonols. The strategy aimed at alleviating the major rate-limiting factor of the tomato flavonoid pathway by upregulating the expression of *CHI* followed by redirection of the pathway towards the anthocyanins. Authors reported a significant increase of both flavonols and anthocyanins in the fruit peel and flesh, reaching up to 200-fold the total flavonol content of wild-type tomatoes (Lim and Li, 2017a). Likewise, Lim and Li (2017b) expressed the *AcCHI* in combination with the *AtPAP1* gene. The combination led to a 130-fold increase in rutin, along with a 30-fold increase of total anthocyanins, compared to the wild-type peel (Lim and Li, 2017b). It is also worth mentioning that the *AcFLS* gene was able to increase rutin content by as much as 3.5-fold in ‘Rubion’ transgenic tomatoes, in contrast to what was observed with the *PhFLS* gene, which was only effective when in combination with *PhCHI* (Verhoeyen et al., 2002; Lim and Li, 2017b). Regarding phenolic acids, the contents of chlorogenic acid, caffeic acid, and coumaric acid were significantly higher in the *AtPAP1*-only lines compared to the wild type, and only modest increases were observed in the gene-stacked lines (Lim and Li, 2017b).

MYB-type TF genes have been successfully used to increase the flavonoid content in tomato. Luo et al. (2008) developed transgenic ‘MicroTom’ and ‘Moneymaker’ plants expressing the *A. thaliana* transcription factor *AtMYB12* and reported strikingly-high amounts of rutin, kaempferol rutinoside, as well as high levels of caffeoylquinic acid and chlorogenic acid, in both fruit peel and flesh. Authors reported that flavonoid content made up to 10% of the fruit dry weight and that it had no significant effect on the carotenoid accumulation of transformed tomatoes (Luo et al., 2008). Zhang et al. (2015) combined *AtMYB12* with *Del*/*Ros1* to generate the Indigo phenotype, exhibiting intense blue-purple-colored fruits. These fruits had a two-fold increase of chlorogenic acid and a three-fold increase of flavonols compared to the *AtMYB12*-carrying line, plus a two-fold increase of anthocyanins compared to the purple *Del*/*Ros1* tomatoes. The combination of TFs was able to activate all the genes encoding enzymes of primary metabolism through to the flavonoid biosynthesis in both the peel and flesh of tomato fruits (Zhang et al., 2015). Likewise, the expression of the *AtMYB12* homolog, *AtMYB11*, in the genetic background of *S. lycopersicum* var. ‘CSL’ led to an increase of both flavonoid and caffeoylquinic acids content (Li et al., 2015). Alterations of the flavonol chemical profile were greater in the fruit peel, and transformed plants accumulated up to 18- and 33-fold the amount of rutin and kaempferol rutinoside, respectively, compared to the controls. In the flesh, flavonols accumulation was modest, showing a 3-fold increase of kaempferol rutinoside compared to the controls (Li et al., 2015).

Wang et al. (2018) developed *SlMYB12*-overexpressing transgenic tomato plants using the cultivars ‘MicroTom’, ‘CSl09-03’ and ‘Sheng Nv-Guo’. This led to a five-fold increase of naringenin chalcone, 15-fold increase of rutin and five-fold increase of kaempferol rutinoside, on average (Wang et al., 2018), in accordance to the findings of Adato et al. (2009). Recently, Wu et al. (2020) developed *SlMYB72*-downregulated transgenic lines of ‘MicroTom’ and reported that total flavonoid content, as well as quercetin and kaempferol derivatives, gallic, and chlorogenic acids content, were significantly increased compared to upregulated plants (Wu et al., 2020).

Both eggplant and pepper are recalcitrant species to *in vitro* organogenesis and therefore the application of genetic engineering techniques has lagged behind compared to tomato (Gammoudi et al., 2017; García-Fortea et al., 2021). In eggplant, a recent work used agroinfiltration for the transient overexpression of *SmHQT* and showed a 2-fold increased CGA content (Kaushik et al., 2020). Using VIGS of *SmCHS* in eggplant cultivar ‘Zhongnong Changfeng’ ripening fruits, Wang and Fu (2018) were able to produce silenced plants showing white peel, resulting from a 2-fold decrease of naringenin and delphinidin. Strikingly, the authors reported an increase of some flavonols and isoflavonoids, compared to mock-inoculated plants, in spite of blocking *SmCHS*. Wang and Fu (2018) hypothesized that chalcone reductase and isoflavone synthase are able to use malonyl-CoA to synthesize isoflavonoids. In relation to the increase of some flavonols, the authors stated that the adoption of an alternative route through caffeoyl-CoA and the mobilization of flavonoids from other tissues may explain this result. The fact that *SmCHS2* kept 31% of its activity after the blocking may also help explain the increase in flavonols (Wang and Fu, 2018). Moreover, authors discussed the involvement of *CHS* in epidermal cell expansion during ripening, after silenced plants yielded uneven, curved and shorter fruits compared to controls. These alterations were also observed at the cellular level, with epidermal cells being compactly arranged, shorter and wider in the white-pigmented sectors compared to the controls. Finally, *CHS*-silenced fruits showed a depressed gravitropic response, implying that either *CHS* or certain flavonoids could be involved in the fruit gravitropic response (Wang and Fu, 2018). This is in agreement to previous findings in tomato (Schijlen et al., 2007; España et al., 2014). Finally, in pepper, application of VIGS targeting MYB and WD40 TFs led to promising results. Aguilar-Barragán and Ochoa-Alejo (2014) showed that the accumulation of anthocyanins in fruits of MYB- and WD40-silenced plants was significantly decreased, compared to the controls. Both transformants showed lower expression of *CHS*, *F3´5´H*, *DFR* and *3GT*. In MYB-silenced plants *CHI* was also negatively affected, whereas in WD40-silenced plants it showed no significant difference. Aversely, *F3H* expression was not affected in MYB-silenced plants while for WD40-transformants it did.

## 4 Potential drawbacks of breeding for increased phenolics

Due to the wide array of functions of phenolic compounds in plant development, and to the interrelationships among the different enzymes and regulatory elements of the phenylpropanoid pathway and with other pathways of plant metabolism, increasing their content in vegetables may affect other traits. That should not be overlooked during the breeding process.

In this respect, some studies have associated increased content of phenolics with lower fruit organoleptic quality. For instance, *SlMYB12* has been reported to act upon genes related to the primary metabolism in tomato (Zhang et al., 2015; Fernandez-Moreno et al., 2016). In this way, the flow of carbon was channeled towards the phenylpropanoid pathway, which indirectly decreased the sugars accumulated in the fruit, which ultimately could affect its flavor. Interestingly, pleiotropic effects related to fertilization and fruit appearance have also been observed in tomato when silencing a structural gene of the flavonoid pathway. Schijlen et al. (2007) blocked *CHS* through RNAi leading to a dramatic decrease of total flavonoid levels in transgenic plants. This was accompanied by a delay of fruit development and smaller parthenocarpic fruits with an altered reddish color and a dull appearance, compared to the wild-type. This shows that flavonoids, despite being considered secondary metabolites, play a fundamental role in different processes of plant reproduction, fruit development and growth.

On the other hand, in eggplant, enzymatic browning occurs when phenolics, which are mostly confined in the vacuole, are released by the rupture of cellular compartments and oxidized by polyphenol oxidases (PPOs), resulting in brown coloration of the flesh, and thus, reduces the apparent fruit quality. That may suggest that increasing the phenolics content in the fruit would lead to higher browning, and indeed, this has been a major concern in eggplant breeding (Plazas et al., 2013; Kaushik et al., 2017). However, a correlation study among browning, phenolics, and PPO activity, casted doubts on a strong relationship between total phenolics or chlorogenic acid contents and fruit flesh browning (Kaushik et al., 2017). Also, several studies have shown that there is variation within the cultivated genepool for selecting varieties with increased phenolics and reduced PPO activity (Mennella et al., 2010; Mennella et al., 2012; Plazas et al., 2013). Another possibility could be through a CRISPR/Cas9-based mutagenesis approach, as mentioned earlier (Maioli et al., 2020).

Phenolic compounds have been associated to astringency and bitterness of foods (Drewnowski and Gomez-Carneros, 2000), which would also be a drawback of increasing phenolics content in tomato, eggplant and pepper. Nevertheless, at the concentrations normally found in these vegetables, it has no appreciable impact on those characteristics, which are mostly caused by saponins, glycoalkaloids and other compounds (Rosa-Martínez et al., 2022a). Also, due to the role of different flavonoids in the fruit cuticle and epidermal cells (España et al., 2014; Heredia et al., 2015), increasing their content could lead to a tougher fruit skin, which may in some cases be unpalatable to the consumer. In any case, pleiotropic effects of the modification of the phenylpropanoid pathway should be well studied as they may prove to be an advantage, like the production of parthenocarpic fruits (Schijlen et al., 2007), or a disadvantage (lower fruit quality) in commercialization of the product.

## 5 Concluding remarks

We present herein a comprehensive review of the literature on the genetics of the phenylpropanoid pathway, as well as on the breeding strategies available to increase their content in the fruits of tomato, eggplant and pepper. We compiled ~267 QTLs linked to phenolic acids, flavonoids and total phenolics content in the three Solanaceae species, along with many candidate genes putatively controlling those QTLs effects. Tomato stands out as the most studied species (217 out of 267 QTLs), a result of its status as model species, whereas eggplant (16 out of 267 QTLs) and pepper (34 out of 267 QTLs) are clearly lagging behind. Notwithstanding, the availability of genomic resources, such as synteny maps and high-quality annotated genomes, has undoubtably contributed to boosting our understanding of the phenylpropanoid pathway and closing the gap among tomato, eggplant and pepper, in spite of the disparity in available genetic resources. Due to their important roles in plant health and development, as well as in the human body, phenolic compounds are gaining interest in breeding programs. We hope that this review facilitates the development of new tomato, eggplant and pepper varieties with improved phenolic profile and greater functional potential. To that end, breeders must take into account not only the qualitative differences among these crops but also the genetic components involved. Hence, structural genes, transcriptional and post-transcriptional regulators, including promoters, repressors, cellular transporters and other types of proteins, should all be taken into account to increase the phenolics content in tomato, eggplant and pepper. Furthermore, the qualitative variations are intrinsically linked to fruit development and are tissue-specific, hence, an accurate understanding on how transcript levels change with these and other factors, would translate into far better results. Identification and analyses of allelic variation of many of the candidate genes governing phenolics accumulation QTLs remains a task at hand, as well as their putative epistatic interactions with other QTLs and genes. Precise genome editing along with next-generation sequencing techniques are important tools towards that end. Ultimately, the development of functional markers could transform the way we improve phenolics content in the fruits of tomato, eggplant and pepper.

## Author contributions

ER-M and LP-D did the literature research, drafted the manuscript and made the tables and figures. AB supervised the literature research and helped to structure the manuscript. JP, MP, YT, and AB provided comments and helped in the writing of the manuscript. All authors contributed to the article and approved the submitted version.
